# Targeted imaging of lysosomal zinc ions with a tetrahedral DNA framework fluorescent reporter

**DOI:** 10.1093/nsr/nwae307

**Published:** 2024-09-12

**Authors:** Yue Gao, Xia Liu, Wei Li, Yuncong Chen, Shitai Zhu, Qinglong Yan, Shanshan Geng, Jichao Zhang, Yong Guan, Qian Li, Sisi Jia, Lihua Wang, Jiang Li, Weijiang He, Chunhai Fan, Zijian Guo, Ying Zhu

**Affiliations:** CAS Key Laboratory of Interfacial Physics and Technology, Shanghai Institute of Applied Physics, Chinese Academy of Sciences, Shanghai 201800, China; Institute of Materiobiology, College of Sciences, Shanghai University, Shanghai 200444, China; University of Chinese Academy of Sciences, Beijing 100049, China; Xiangfu Laboratory, Jiaxing 314102, China; CAS Key Laboratory of Interfacial Physics and Technology, Shanghai Institute of Applied Physics, Chinese Academy of Sciences, Shanghai 201800, China; University of Chinese Academy of Sciences, Beijing 100049, China; State Key Laboratory of Coordination Chemistry, School of Chemistry and Chemical Engineering, Nanjing University, Nanjing 210023, China; CAS Key Laboratory of Interfacial Physics and Technology, Shanghai Institute of Applied Physics, Chinese Academy of Sciences, Shanghai 201800, China; University of Chinese Academy of Sciences, Beijing 100049, China; Xiangfu Laboratory, Jiaxing 314102, China; State Key Laboratory of Coordination Chemistry, School of Chemistry and Chemical Engineering, Nanjing University, Nanjing 210023, China; Shanghai Synchrotron Radiation Facility, Shanghai Advanced Research Institute, Chinese Academy of Sciences, Shanghai 201210, China; National Synchrotron Radiation Laboratory, University of Science and Technology of China, Hefei 230026, China; School of Chemistry and Chemical Engineering, New Cornerstone Science Laboratory, Frontiers Science Center for Transformative Molecules and National Center for Translational Medicine, Shanghai Jiao Tong University, Shanghai 200240, China; Zhangjiang Laboratory, Shanghai 201210, China; CAS Key Laboratory of Interfacial Physics and Technology, Shanghai Institute of Applied Physics, Chinese Academy of Sciences, Shanghai 201800, China; Institute of Materiobiology, College of Sciences, Shanghai University, Shanghai 200444, China; University of Chinese Academy of Sciences, Beijing 100049, China; CAS Key Laboratory of Interfacial Physics and Technology, Shanghai Institute of Applied Physics, Chinese Academy of Sciences, Shanghai 201800, China; Institute of Materiobiology, College of Sciences, Shanghai University, Shanghai 200444, China; University of Chinese Academy of Sciences, Beijing 100049, China; State Key Laboratory of Coordination Chemistry, School of Chemistry and Chemical Engineering, Nanjing University, Nanjing 210023, China; School of Chemistry and Chemical Engineering, New Cornerstone Science Laboratory, Frontiers Science Center for Transformative Molecules and National Center for Translational Medicine, Shanghai Jiao Tong University, Shanghai 200240, China; State Key Laboratory of Coordination Chemistry, School of Chemistry and Chemical Engineering, Nanjing University, Nanjing 210023, China; CAS Key Laboratory of Interfacial Physics and Technology, Shanghai Institute of Applied Physics, Chinese Academy of Sciences, Shanghai 201800, China; Institute of Materiobiology, College of Sciences, Shanghai University, Shanghai 200444, China; University of Chinese Academy of Sciences, Beijing 100049, China

**Keywords:** DNA framework (DNF), tetrahedron, lysosome, zinc ions, Alzheimer's disease

## Abstract

Abnormal levels of zinc ions within endo-lysosomes have been implicated in the progression of Alzheimer's disease (AD), yet the detection of low-concentration zinc ions at the organelle level remains challenging. Here we report the design of an endo-lysosome-targeted fluorescent reporter, Znluor_ly_, for imaging endogenous zinc ions. Znluor_ly_ is constructed from an amphiphilic DNA framework (DNF) with programmable size and shape, which can encapsulate zinc-responsive fluorophores within its hydrophobic nanocavity. We find that the tetrahedral DNFs of 20 bp in the edge length are effectively located within endo-lysosomes, which can detect zinc ions with a detection limit of ∼31.9 nM (a sensitivity that is ∼2.5 times that of the free fluorophore). Given the organelle-targeting ability and high zinc sensitivity of Znluor_ly_, we employ it to detect endogenous endo-lysosomal zinc ions in neuron cells. We monitor the dynamics of zinc levels in AD model cells and zebrafish, corroborating the positive correlation between zinc levels and AD hallmarks including Aβ aggregates and learning/memory impairments. Our study provides a generalizable strategy for organelle-specific theranostic applications.

## Teaser

A DNA-based fluorescent reporter for endo-lysosomal zinc ions, named Znluorly, is developed. Its application potential is demonstrated in monitoring zinc dynamics in Alzheimer's disease model cells and zebrafish.

## INTRODUCTION

Dyshomeostasis of zinc is pivotal in the advancement of Alzheimer's disease (AD) [1]. Endo-lysosomes, the primary organelles storing endogenous zinc ions (Zn^2+^), when overloaded, may trigger lysosomal membrane permeabilization (LMP) [2], leading to the release of various acid hydrolases into the cytosol. This event potentially results in the formation of Aβ aggregates [2,3]. Hence, Zn^2+^ can serve as a marker for monitoring AD progression. However, the endogenous Zn^2+^ concentrations in neuronal cells typically fall below the detection thresholds of current *in-situ* imaging techniques [4]. This necessitates the development of a sensitive probe for imaging endo-lysosomal Zn^2+^ in neuronal cells.

Over recent decades, substantial effort has been dedicated to the development of Zn^2+^-sensitive probes [5–12]. Yet few have enabled the detection of endogenous endo-lysosomal Zn^2+^ in living cells, largely due to trade-offs in the areas of hydrophilicity, brightness, endo-lysosomal targeting and Zn^2+^ sensitivity. For example, organic dyes, typically hydrophobic small molecules [13], require covalent chemical modifications for water solubility and organelle-specific targeting, which may compromise their brightness and thus sensitivity. Larger-sized inorganic nanoparticles, such as quantum dots, metal nanoclusters and upconversion nanoparticles, often exhibit strong and stable brightness [14–17], and some have shown the ability to detect Zn^2+^  *in vitro* or when externally introduced into cells [18–20]. Nonetheless, the detection of endogenous Zn^2+^ in endo-lysosomes continues to pose a significant challenge [21–23].

**Figure 1.**
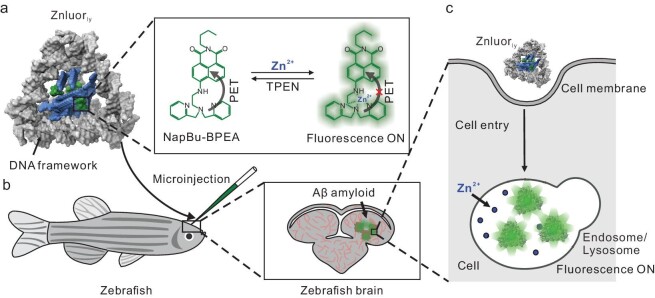
Schematic illustration of Znluor_ly_ for endo-lysosome-targeted imaging of zinc ions. (a) Structure and chemical principle of Znluor_ly_. (b) Application of Znluor_ly_ in imaging endogenous Zn^2+^ in neural cells of the AD model zebrafish. (c) Principle of the endo-lysosome-targeting ability of Znluor_ly_.

The advancement of DNA nanotechnology has facilitated the fabrication of DNA framework structures (DNFs) characterized by programmable geometric parameters, superior hydrophilicity and biocompatibility [24]. These structures can act as universal scaffolds for the precise spatial arrangement of various functional materials such as fluorescent dyes [25], inorganic nanoparticles [26] and proteins [27]. Notably, DNFs can be internalized by mammalian cells and localized in endo-lysosomes, enabling their use as targeted carriers for these organelles [28,29]. Recently, DNFs integrated with target-selective fluorescent reporters have been investigated as imaging probes for detecting specific ions or molecules within endo-lysosomes [28–33]. In this study, we encapsulated a Zn^2+^-responsive fluorescent organic molecule into a DNF with a hydrophobic nanocavity, creating a Zn^2+^ fluorescent reporter, Znluor_ly_, for imaging endogenous Zn^2+^ in living cells’ endo-lysosomes (Fig. 1). We examined the influence of the DNF's size and shape on its endo-lysosomal targeting ability. We also assessed the probe's chemostability, and sensitivity to Zn^2+^ in physiological environments. As a demonstration, we used Znluor_ly_ for extended tracking of endogenous endo-lysosomal Zn^2+^ in AD model cells and zebrafish.

## RESULTS

### Design, synthesis and characterization of Znluor_ly_

**Figure 2.**
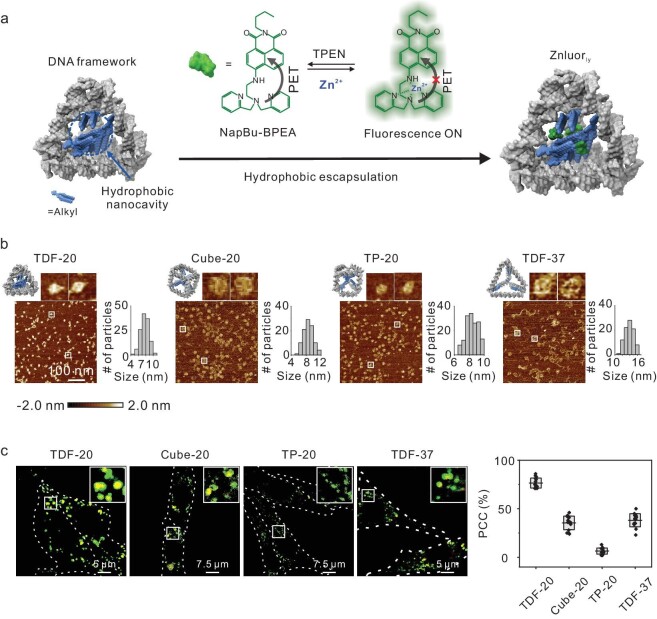
Design of Znluor_ly_ and characterization of DNA frameworks (DNFs). (a) Scheme for the design and synthesis of Znluor_ly_. (b) Atomic force microscopic (AFM) images and size distribution statistics (*n* > 90) of different DNFs. Scale bar = 100 nm. The experiments were repeated three times independently. (c) Colocalization of different DNFs with lysosomes. The colocalization of red and green fluorescent signals in cells was quantified using Image J software. Data are presented individually and as the mean ± S.D. (center line with error box) of *n* = 15 for each group.

We first describe the design and characterization of Znluor_ly_ (Fig. 2a). A Znluor_ly_ reporter is composed of two modules: (i) a Zn^2+^ reporter module and (ii) a module for molecular encapsulation and endo-lysosome targeting. Module 1 is a previously described Zn^2+^-sensitive molecule, NapBu-BPEA [34] (Figs S1–S4). In this molecule, *N,N*′-bis(pyridin-2-ylmethyl) ethane-1,2-diamine (BPEA) is an electron-rich moiety that can specifically chelate Zn^2+^ while *N*-*n*-butyl-1,8-naphthalimide (NapBu) serves as a fluorophore. In the absence of Zn^2+^, the photoinduced electron transfer (PET) between BPEA and NapBu would suppress the fluorescence emission of NapBu. However, when Zn^2+^ is chelated by BPEA, the PET process is inhibited, resulting in the fluorescence ‘turn-on’. Despite its functionality, this organic molecule's lack of sufficient water solubility and endo-lysosome-targeting ability limits its effectiveness in detecting endogenous endo-lysosomal Zn^2+^ in living cells. Module 2, on the other hand, is a DNF structure of defined size and shape, assembled from multiple single-stranded (ss-) DNAs. Some ssDNAs, terminated with dendritic alkyl chains, can form a hydrophobic cavity within the hydrophilic framework in water solution, as previously described [35]. We anticipated that this DNF module could non-covalently encapsulate the probe molecule NapBu-BPEA via hydrophobic interaction, facilitating the hydrophobic-to-hydrophilic conversion of the probe with minimal disruption to its chemical/optical properties.

In pursuit of a DNF structure with optimal endo-lysosome-targeting ability, we synthesized and evaluated four distinct DNFs of varying shapes and sizes, as adapted from previous literature (Fig. 2b). These included a tetrahedron [35–37], a cube and a triangular prism, all with an edge length of 20 basepairs (bp) (designated as TDF-20, Cube-20 and TP-20), along with a larger tetrahedron with an edge length of 37 bp (TDF-37). Each DNF was assembled separately with component ssDNAs, some of which were terminated with dendritic alkyl chains (detailed in Supplementary Methods), using a thermal annealing process described elsewhere. Native polyacrylamide gel electrophoresis (PAGE) analyses (Fig. S5) yielded distinct bands for the DNFs, with migration rates aligning with expectations based on the markers. AFM images revealed near-monodispersed particle-like structures with apparent sizes of ∼7.8, 8.6, 8.4 and 13.9 nm, corresponding to TDF-20, Cube-20, TP-20 and TDF-37, respectively (Fig. 2b). These results confirmed the successful formation of these DNFs.

Next, we incubated these DNF structures (labeled with Cy5) with PC12 cells (a rat pheochromocytoma cell line commonly employed as a neurobiology model) for 2 h. Post-incubation, TDF-20, among the tested structures, exhibited the highest red fluorescence intensity within the cells (Fig. 2c and Fig. S6), suggesting its superior cell uptake efficiency. The fluorescent puncta of DNFs were predominantly colocalized with lysosomes. However, the Pearson's colocalization coefficients (PCCs) [38] between the DNFs’ fluorescent puncta and those from the lysosome dye (LysoTracker Green) were ∼76% ± 5%, 35% ± 7%, 6% ± 3% and 38% ± 7% for TDF-20, Cube-20, TP-20 and TDF-37, respectively. This suggests that the lysosomal occupancies by the DNFs varied due to differences in cellular uptake efficiencies, which are dependent on their shape and size. In this context, a higher PCC value indicates a greater lysosomal occupancy by the DNF structures, suggesting a stronger lysosome-targeting capacity. In summary, while all DNF structures exhibited some degree of lysosome-targeting ability, TDF-20 displayed the highest efficiency due to its superior cellular uptake efficiency. Consequently, TDF-20 was selected as the DNF module for subsequent construction of Znluor_ly_.

**Figure 3.**
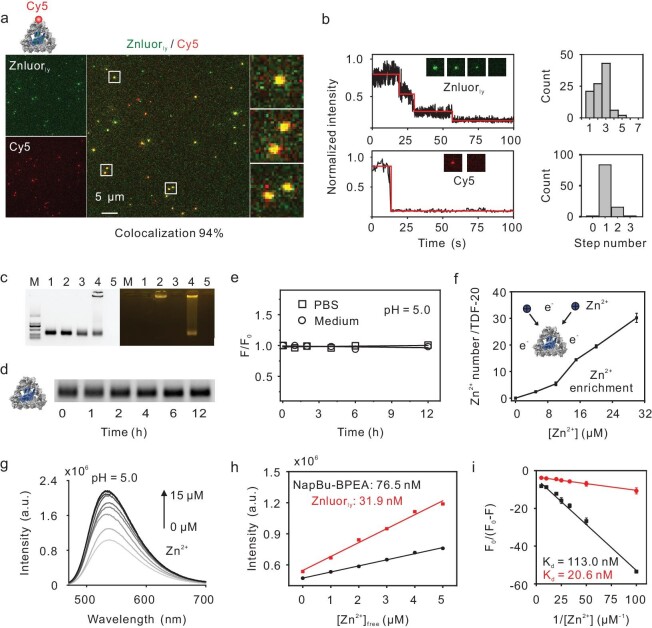
Optical characterization of Znluor_ly_. (a) Single-molecule fluorescence images and photobleach experiment of Znluor_ly_ dispersed in solution. Scale bar: 5 μm. (b) Intensity traces of Znluor_ly_ modified with Cy5 (left) and distributions of quenching step counts (*n* = 100) (right). (c) Agarose gel (1% w/v) electrophoresis image of Znluor_ly_. Left, image under UV illumination (DNFs were stained with GelRed); right, image under blue illumination (fluorescence from NapBu-BPEA). Lane 1 to 5, alkyl-free DNFs, alkyl-free DNFs incubated with NapBu-BPEA, alkyl-DNFs, Znluor_ly_ (alkyl-DNFs incubated with NapBu-BPEA) and free NapBu-BPEA, respectively. (d) Gel electrophoresis image of TDF-20 structures incubated in the medium with 10% fetal bovine serum (FBS) for 0–12 h. (e) Fluorescence intensity of Znluor_ly_ versus incubation time in physiological environments at pH 5.0. (f) Enrichment of Zn^2+^ by TDF-20. TDF-20 was incubated with Zn^2+^ of different concentrations, separated via ultrafiltration, and then underwent inductively coupled plasma mass spectrometry measurement of Zn^2+^ adsorbed on these structures. Data are presented as mean ± S.D. (*n* = 3 independent tests). (g) Emission spectra of Znluor_ly_ (1.6 μM, pH 5, *λ*_ex_ 450 nm) in response to Zn^2+^ of different concentrations (0, 1, 2, 3, 4, 5, 8, 10 and 15 μM, respectively) in the solution (10 mM Tris-HCl, 5 mM MgCl_2_, pH 5.0). (h) Linear fittings and limits of detection (LOD) of Znluor_ly_ (1.6 μM, equivalent to 5 μM of NapBu-BPEA) and free NapBu-BPEA (5 μM). Red line, linear fitting of the Zn^2+^ responses of Znluor_ly_, *y* = 134492.1*x* + 546672.8 (*R*^2^ = 0.991); black line, linear fitting for free NapBu-BPEA, *y* = 58890.2*x* + 472832.4 (*R*^2^ = 0.996). (i) Dissociation constants (*K*_d_) of Znluor_ly_ and NapBu-BPEA. F_0_ represents the initial fluorescence intensity of free Znluor_ly_ (1.6 μM) and NapBu-BPEA (5 μM) in TM buffer (10 mM Tris-HCl, 5 mM MgCl_2_, pH 5.0). F represents the observed fluorescence intensity at its maximum. The concentrations of Zn^2+^ range from 0.01 to 0.2 μM. Data are presented as mean ± S.D. (*n* = 3 independent tests).

We synthesized Znluor_ly_ via the molecular encapsulation of NapBu-BPEA with TDF-20, facilitated by hydrophobic interactions. This process was achieved by mixing the two components in an aqueous solution (detailed in Methods). Single-molecule imaging revealed a high degree of colocalization (colocalization coefficient ∼94%) between the fluorescent signals of NapBu-BPEA and Cy5-labeled TDF-20 (Fig. 3a). This colocalization was further confirmed by the agarose gel image, which showed the overlapping of the NapBu-BPEA fluorescence with the TDF-20 band (Fig. 3c). In contrast, the mixture of NapBu-BPEA and alkyl-free DNFs exhibited no discernible fluorescent band at the position of the DNFs’ band (lane 2 in Fig. 3c). This indicates that there was no significant binding between the alkyl-free DNFs and NapBu-BPEA. Once we employed Cy3-labeled TDF-20 for encapsulation, a prominent Förster resonance energy transfer (FRET) effect could be observed between NapBu-BPEA and Cy3 (Fig. S7), indicating their spatial proximity and thus, successful encapsulation. Analysis of fluorescence photobleaching indicated one to five photobleaching steps per fluorescent spot, with three being the highest frequency (Fig. 3b). This suggests that each TDF-20 structure could accommodate ∼3 NapBu-BPEA molecules.

We further evaluated the contribution of TDF-20 encapsulation to the fluorescence intensity in the aqueous solution. Without TDF-20 encapsulation, the free NapBu-BPEA molecules (1–10 μM) showed significantly lower fluorescence intensities in an aqueous solution (Tris-MgCl_2_ (TM) buffer) (Fig. S8) than in dimethyl sulfoxide (Fig. S9, lower left). This could be attributed to the aggregation-caused quenching (ACQ) effect. In comparison, the encapsulation by TDF-20 resulted in remarkably enhanced brightness with the equivalent dye concentration (Fig. S9, lower right), suggesting that encapsulation can effectively mitigate ACQ of the dye molecules in aqueous environments.

Having synthesized the Znluor_ly_ structure, we next tested its stability in physiological environments. After 12-h incubation with the complete cell culture medium (containing 10% v/v bovine serum), the gel band of Znluor_ly_ remained almost unchanged (Fig. 3d and Fig. S10c). In comparison, a double-stranded DNA (dsDNA) structure decayed significantly in 6 h (Fig. S10). These results suggest the superior structural stability of DNF against nuclease degradation in physiological environments as compared to dsDNA, in agreement with previous studies [39,40]. We also found that after a 12-h period, the fluorescence intensities of Znluor_ly_ in phosphate-buffered saline (PBS)/cell culture medium diminished by 4.0%/1.7% at pH 5.0 and 4.0%/2.5% at pH 7.0, respectively. These results suggest that Znluor_ly_ is stable in both physiological and acidic conditions (Fig. 3e and Fig. S11).

### Responsiveness of Znluor_ly_ to Zn^2+^

To assess the Zn^2+^ responsiveness of Znluor_ly_, we performed titration experiments in an acidic medium (pH 5.0), simulating the lysosomal environment. Upon the introduction of Zn^2+^ into a Znluor_ly_’s solution (5 μM), we observed a positive correlation between the concentration of Zn²⁺ and its adsorption onto TDF-20 (Fig. 3f), suggesting Zn^2+^ enrichment by the negatively charged DNFs via electrostatic interactions. The fluorescence emission intensity of Znluor_ly_ (peak at 535 nm, excited at 454 nm) progressively increased with Zn^2+^ concentration (0, 1, 2, 3, 4, 5, 8, 10 and 15 μM) at pH 5.0, plateauing at ∼8 μM (Fig. 3g and Fig. S12). The limit of detection (LOD) and dissociation constant (*K*_d_) of Znluor_ly_ for Zn^2+^ were estimated to be ∼31.9 nM and 20.6 nM, respectively (Fig. 3h and i). In contrast, the LOD and *K*_d_ of free NapBu-BPEA molecules were ∼76.5 nM and 113.0 nM, respectively. The encapsulation of fluorescent molecules within the TDF structure resulted in a ∼2.5-fold enhancement in Zn^2+^ sensitivity. This enhanced sensitivity can be attributed to two key factors: (i) the Zn^2+^ enrichment effect induced by TDF, which resulted in a high local Zn^2+^ concentration surrounding NapBu-BPEA; (ii) the hydrophobic nanocavity in TDF of a defined size, encapsulated a limited number of NapBu-BPEA molecules, resulting in good dispersion in the aqueous medium and thus high fluorescence brightness.

We further examined the specificity of Znluor_ly_ in the presence of other metal ions. Despite the 1000-fold higher concentration (2 mM) of Na^+^, K^+^, Ca^2+^ and Mg^2+^—ions known to be abundant in cells—only negligible fluorescence was observed, compared to the positive control of Zn^2+^ (2 μM). Similarly, the presence of Ag^+^, Fe^2+^, Hg^2+^, Ni^2+^, Mn^2+^, Co^2+^, Cd^2+^, Cu^2+^ and Pb^2+^ (2 μM) did not elicit significant fluorescence relative to Zn^2+^ at the same concentration (Fig. S13). These findings underscore the specificity of Znluor_ly_ for Zn^2+^.

### Measurements of endogenous endo-lysosomal Zn^2+^ in living cells

**Figure 4.**
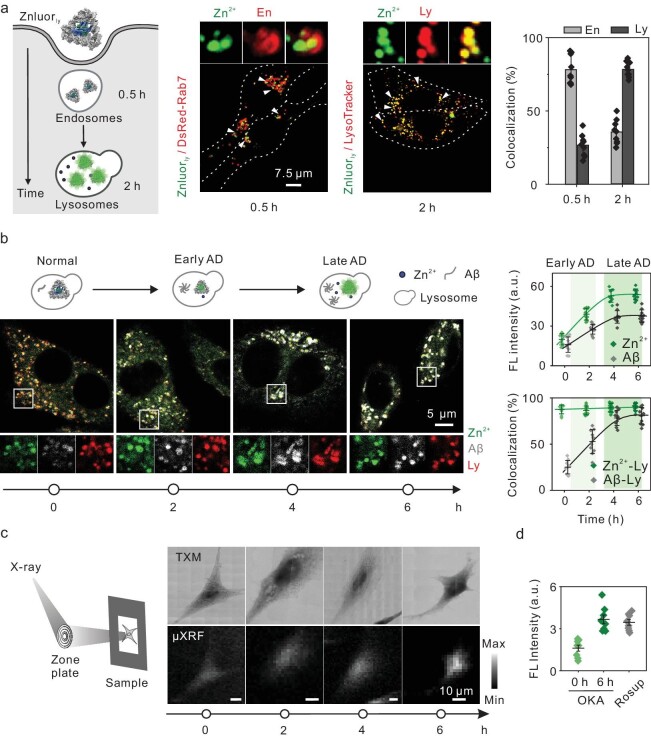
Znluor_ly_ enables measurement of endogenous lysosomal Zn^2+^ in living cells. (a) Trafficking of Znluor_ly_ in PC12 expressing DsRed-Rab7 (left) or LysoTracker (right) at the indicated times. En: endosome; Ly: lysosome. Scale bars, 7.5 μm. Error bars, mean ± S.D. (column with error bar) of *n* = 9 cells. (b) PC12 cells were pre-treated with 100 nM okadaic acid (OKA) for various periods, and then stained with Znluor_ly_ (300 nM), thioflavin T (Th T, 300 nM) and LysoTracker Red (a commercially available marker for lysosomes; 1 : 20 000) for 2 h. Left: confocal images of cells. Right: fluorescence intensity inside the cells and colocalization efficiency of Zn^2+^ with lysosomes, as well as Aβ with lysosomes. Pseudocolored representative images were generated using Image J. Data are presented individually (colored dots) and as the mean ± S.D. (center line with error bar) of *n* = 10 for each group. Scale bar: 5 μm. (c) μXRF-based imaging of zinc element within AD cells. Scale bar: 10 μm. (d) Reactive oxygen species (ROS) generation measured using 2′,7′-dichlorodihydrofluorescein diacetate (DCFH-DA). Data are shown individually (colored dots) and as mean ± S.D. (center line with error bar) of *n* = 8 pictures.

We proceeded to use Znluor_ly_ to track endogenous Zn^2+^ within endo-lysosomes. We first employed normal PC12 cells as the model, which were transfected with DsRed-Rab7 or pre-loaded with LysoTracker. Time-resolved fluorescence imaging revealed that the fluorescent signals of Znluor_ly_ were predominantly colocalized with endosomes at 30 min post-incubation, and subsequently with lysosomes at 2 h post-incubation (Fig. 4a and Figs S14 and S15), confirming that Znluor_ly_ can target endo-lysosomes in living neuron-like cells, in agreement with the previously described mechanism for cellular uptake of nanomaterials [30,41]. In addition, thiazolyl blue assay results indicated that Znluor_ly_ exhibited minimal toxicity to cells after a 12-h incubation, even at the maximum exposure dose of 600 nM (Fig. S16).

To verify whether the signal intensity of Znluor_ly_ can indicate Zn^2+^ level in endo-lysosomes, we challenged Znluor_ly_ with AD model cells, whose intracellular Zn^2+^ levels would be higher than normal cells [1]. We first prepared AD model cells by treating normal PC12 cells with okadaic acid (OKA), a protein phosphatase 2A inhibitor that can induce neurodegeneration [42,43]. We found that with the increase in OKA treatment time, the levels of Aβ-fragments and p-tau protein (a pathological hallmark of AD) increased correspondingly (Fig. S17) [44–47], suggesting the successful establishment of pharmacologically induced AD model cells.

Next, we subjected Znluor_ly_ to AD model cells at various stages. We found that the fluorescence intensities increased with the rise of Aβ level (labeled with Aβ antibody) (Fig. 4b), indicating a positive correlation between the signal intensity and AD progression. In addition, control studies using free NapBu-BPEA dyes for AD model cells showed significantly lower fluorescence intensities than Znluor_ly_ (Fig. S18). We further employed synchrotron-based micro X-ray fluorescence (μXRF) microscopy to map the zinc element in the AD cells (Fig. 4c and Fig. S19), confirming an increase in intracellular zinc level in line with AD progression, consistent with the fluorescence results. Moreover, the fluorescence colocalization efficiencies between Aβ, and lysosomes raised along with AD progression, reached a plateau in the late stage of AD (OKA treatment for >4 h) (Fig. 4b and Fig. S20), in agreement with previous findings that Aβ deposits accumulate in lysosomes with the development of AD [3,48]. Meanwhile, ∼90% of Znluor_ly_ signals were constantly colocalized with lysosomes. Additionally, we discerned a positive correlation between the Znluor_ly_ signal intensity and the intracellular ROS level, another hallmark of AD progression (elevated reactive oxygen species (ROS) levels are characteristic of late-stage AD) [49,50] (Fig. 4d). Collectively, we conclude that Znluor_ly_ can effectively indicate the level of endogenous Zn^2+^ in the endo-lysosomes of AD model cells.

### Znluor_ly_ enables Zn^2+^ tracking in the brain of AD model zebrafish

**Figure 5.**
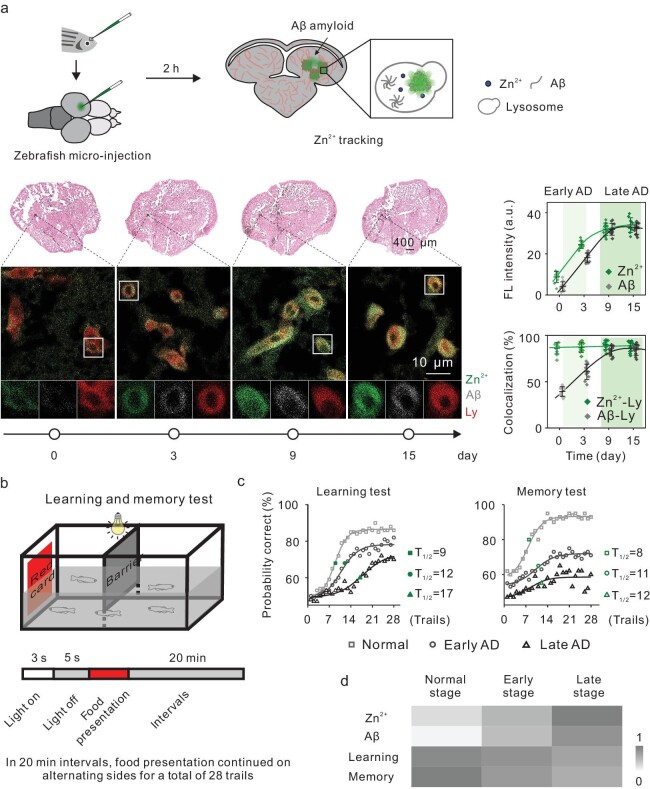
Zn^2+^ tracking in the brain of AD model zebrafish. Zebrafish were pre-incubated with OKA (200 nM) for various lengths of time. (a) Zebrafish were injected with Znluor_ly_ for 2 h (300 nM) and then the brain slice sections were obtained and stained with Aβ antibody (1 : 1000) and LAMP1 for 1 h. Left: confocal images of brain sections. Right: the fluorescence intensity per cell. Data are presented individually (colored dots) and as the mean ± S.D. (center line with error bar), *n* = 10 for each group, for Zn^2+^ or Aβ in brain sections and the colocalization efficiency between Zn^2+^ and lysosomes, as well as Aβ and lysosomes. Pseudocolored representative images were generated using Image J. Scale bar: 400 μm. (b) General experimental design for the learning and memory tests of AD zebrafish. (c) Learning and memory data of AD zebrafish. The dots in each group represent the group's running average at each trial point. The curves represent non-linear least-squares regressions of the correct response probabilities. (d) Correlations among Zn^2+^ signal intensity, Aβ protein accumulation and the cognitive ability of zebrafish.

As a proof of concept, we employed Znluor_ly_ to track the spatiotemporal distribution of Zn^2+^ in the brain of AD model zebrafish (*Danio rerio*). We first prepared the pharmacologically induced AD zebrafish model by using OKA [51,52]. After the OKA treatment, we observed microbleeds in the brains (Fig. S21), as well as significantly altered levels of Aβ and p-tau protein (Fig. 5a and Fig. S22), confirming the establishment of the AD model. At given time points after the AD modeling, the zebrafish were injected with Znluor_ly_ via microinjection into the optic tectum [53,54]. Two hours post-injection, the brains were sliced and labeled with Aβ antibody and lysosome-associated membrane protein 1 (LAMP1, a lysosome marker [55,56]). We observed that the Znluor_ly_ signal intensity in brain cells significantly increased 3 days after OKA treatment (Fig. 5a), along with the rising of the Aβ deposit level. Meanwhile, ∼90% of Znluor_ly_ signals were constantly colocalized with lysosomes. The colocalization between Aβ deposits and lysosomes also increased gradually with the development of AD, and reached a plateau in the late stage of AD (Fig. 5a and Fig. S23). These results are in agreement with those from AD model cells, confirming that Znluor_ly_ can be used to study AD animal models.

Finally, we studied the correlation between the pathological change of Zn^2+^ level and learning/memory impairments of the AD zebrafish. We first constructed three groups of AD model zebrafish with varied OKA treatment durations (3, 9 and 15 days, respectively). We accessed the learning and memory function of the zebrafish by using a spatial alternation task for a food reward [57]. All zebrafish were trained in 2-gallon testing tanks for a period of 28 trials (Fig. 5b). During the whole experiment, all groups showed an increase in the number of correct responses at the choice step of food presentation. We observed significant improvement in response accuracy (over the 50% benchmark of random responses) after training. For normal zebrafish, the maximum response accuracy after multiple rounds of training (maximum learning accuracy) was ∼88%, and the half-maximal learning point was at the ninth trial. The maximum learning accuracy of zebrafish presented a time-dependent decrease, which was 82% and 72% corresponding to 3-day and 9-day OKA treatment, respectively. Moreover, AD zebrafish took more trials to reach the half-maximal learning points (12th and 17th trials corresponding to 3-day and 9-day OKA treatment) than normal zebrafish, confirming the decreased learning capability associated with AD progression (Fig. 5c and Fig. S24).

For the evaluation of the memory ability of AD model zebrafish, all zebrafish were removed from the testing tanks and fed *ad libitum* for 10 days. Then they were reintroduced to the testing tank to initiate memory testing for the spatial alternation task. All zebrafish were also trained for a period of 28 trials. For normal zebrafish, they started the test with 62% response accuracy. The maximum learning was ∼92.6% and the half-maximal learning was the eighth trial from the starting point of 62%. In contrast, in the groups treated with OKA for 3 days and 9 days, the initial accuracies for the memory test were 53% and 47%, respectively; the maximum learning accuracies were 71.5% and 59.5%, respectively; the half-maximal learning points were the 11th and 12th trials, respectively (Fig. 5c and Fig. S24). Taken together, these data verify the impaired learning/memory ability of AD zebrafish. More importantly, we have established a positive correlation between the fluorescence signal of endo-lysosomal Zn^2+^ and Aβ protein, as well as a negative correlation between the signal and the zebrafish's learning/memory ability (Fig. 5d). These results further confirm that the endo-lysosomal Zn^2+^ level is correlated with the AD stage-dependent behaviors of animals. Thus, the use of Znluor_ly_ as an indicator of AD progression may benefit related studies.

## DISCUSSION AND CONCLUSIONS

In this work, we described a DNF-based endogenous lysosomal Zn^2+^ reporter, Znluor_ly_, which achieved hydrophobic-to-hydrophilic conversion of the Zn^2+^ sensitive fluorophore via non-covalent encapsulation into the nanocavity of the DNF. Znluor_ly_ showed super brightness, good hydrophilicity and endo-lysosome targeting ability. By using Znluor_ly_, we demonstrated the visual detection of endogenous Zn^2+^ in AD model cells and zebrafish.

This system presents multiple benefits. Firstly, DNFs exhibit high monodispersity in aqueous solutions, and each DNF features a defined hydrophobic nanocavity, allowing non-covalent encapsulation of approximately three organic dye molecules within the nanocavity. In contrast, regular PEGylated (the covalent binding of polyethylene glycol to another molecule) nanocarriers often lack such precision in encapsulation, which may lead to broad distributions in fluorophore number and brightness [58]; however, direct covalent PEGylation of the dye molecules may interfere with their fluorescence, according to previous studies [59,60].

Second, Znluor_ly_ exhibits effective internalization by neuronal cells and localizes in endo-lysosomes providing endo-lysosome targeting capability. This allows for maximum accumulation in lysosomes while minimizing distribution in other subcellular regions. Consequently, the utilization of the probes is maximized, and negative impacts on other regions are reduced. This property enables sensitive monitoring of endogenous lysosomal Zn^2+^ in AD model cells and zebrafish (Fig. 4b and Fig. 5a).

Third, the compact DNF structures exhibit greater stability than linear DNA molecules against enzymatic degradation in physiological environments [39,40]. Finally, the negatively charged DNA shell can help enrich cationic Zn^2+^ via electrostatic attraction, enhancing the sensitivity compared to free dye molecules, a property not present in the nanoparticle shell formed by encapsulation methods such as PEG [61]. These features in combination facilitate sensitive mapping of endogenous endo-lysosomal Zn^2+^ in living cells and zebrafish without additional Zn^2+^. In comparison, although dsDNA molecules have also been utilized for endo-lysosome-targeted molecular imaging [29,62], they lack some aforementioned advantages. On the other hand, in the AD zebrafish models, we correlated the AD development stages with the quantitative, spatial mapping of lysosomal Zn^2+^ in the brain, which may have implications for early diagnosis and treatment of AD in the future. More generally, such DNFs may encapsulate diverse fluorescence dyes in the nanocavity, allowing endo-lysosome-targeted imaging of different molecules, thus facilitating the study and treatment of endo-lysosome-related diseases.

## MATERIALS AND METHODS

### Preparation of NapBu-BPEA

The chemical structure and synthetic route of NapBu-BPEA are detailed in Supplementary Methods (Fig. S1). In brief, 4-bromo-1,8-naphthalic anhydride was treated with *n*-butylamine to yield compound **1**, the Br of which was then substituted by the ethylenediamine to yield compound **2**. Finally, the primary amine group of compound **2** was converted to tertiary amine by reaction with picolyl chloride in the presence of K_2_CO_3_, and yielded NapBu-BPEA [34].

### Preparation and characterization of Znluor_ly_

TDF-20 structures were prepared as previously reported [35–37]. In brief, four single-chain alkyl DNA strands were mixed in equimolar ratio in TM buffer (10 mM tris, 5 mM MgCl_2_, pH 8.0). The solution was heated to 95°C for 15 min and then quickly cooled to 4°C.

TDF-20 structures were purified using high-performance liquid chromatography (HPLC). After purifying TDF-20, NapBu-BPEA (50 μM) was intercalated into TDF-20 (500 nM), via simple incubation overnight, to form Znluor_ly_. Excess NapBu-BPEA was removed through Nap-5 columns. The sequences of TDF-20 are listed in Table S1.

Znluor_ly_ structures were analyzed using gel electrophoresis and AFM. In brief, Native PAGE was performed in 1X TAE (Tris-acetic acid-EDTA) buffer (4 mM Tris base, 2 mM acetic acid, 0.2 mM ethylene diamine tetraacetic acid) at 4°C for about 1–1.5 h. Then gel was stained with GelRed for ∼15 min for evaluation purposes. PAGE analyses confirmed the successful formation of different-shaped DNA frameworks (Fig. S5).

For AFM measurement, 10 μL, 0.5% (3-aminopropyl) triethoxysilane was added to the mica substrate for 1 min to modify the mica surface. After that, Znluor_ly_ or a TDF-20 sample (5 μL) was left to adsorb on the mica surface. 50 μL 1× TAE buffer was added prior to sample scanning under tapping mode using a J-scanner from Multi-mode Nanoscope IIIa AFM (Vecco/Digital Instruments), coupled with a silicon nitride cantilever with sharp pyramidal tip (OMCL-TR400PSA, Olympus). Hydrodynamic size was analyzed with Zeta sizer nano Z (Malvern) by dispersing 1 μM of Znluor_ly_ or TDF-20 in 1 mL distilled water. The measurement was repeated three times.

### Cell culture and treatment

PC12 cells were grown in RPMI1640 (Gibco) cell culture medium supplemented with 10% FBS, and the resultant cell suspension (8 × 10^4^ cells mL^−1^) was dispensed into a confocal dish and incubated overnight to allow for cell adherence. After washing twice with PBS, cells were exposed to Znluor_ly_ (300 nM), LysoTracker Green/Red (1 : 20 000 dilution) and Th T (300 nM) for 60 min at 37°C. The fluorescence imaging was collected by confocal fluorescence microscopy.

### AD models and treatment

To build the cell model of AD at different stages, PC 12 cells were exposed to OKA (100 nM) for 0, 2, 4 and 6 h, respectively, and then were washed three times with PBS. The treated cells were collected for western blotting analysis to verify the establishment of the AD model.

To verify the Znluor_ly_’s ability to detect Zn^2+^ at the *in-vivo* level, we injected the probe into zebrafish brain tissue by microinjection. Zebrafish (12-month) were exposed to OKA (200 nM) in tanks for 9 days to obtain AD zebrafish models (mortality 10%–20%). The water with OKA was refreshed every other day. At the end of day 9, the fish was anesthetized in a final concentration of 0.016% (v/v) TMS (tricaine methane sulfonate) in a petri dish. A fine needle with sharp edges was used to puncture a small hole ∼200 μm in the fish's skull without damaging brain tissue. A maximum of 500 null of Znluor_ly_ was injected using thin glass capillaries. When the zebrafish woke up, they were sacrificed after an hour and placed in 4% paraformaldehyde overnight at 4ºC. Fish brains were dissected and stored at −80ºC. Frozen brains were cut into 6 μm sections and then used in fluorescent imaging [51].

### Evaluation of learning/memory function in zebrafish

The learning and memory behaviors of zebrafish were tested according to a previous report [57]. Zebrafish were put into two-gallon testing tanks (Fig. 5b). A correct response was considered to be the physical presence of the animal on the side of the tank used for food presentation during that trial. 28 trials were recorded and correct responses were compiled for averages and statistical purposes.

## Supplementary Material

nwae307_Supplemental_File

## References

[1] Corona C, Pensalfini A, Frazzini V et al. New therapeutic targets in Alzheimer's disease: brain deregulation of calcium and zinc. Cell Death Dis 2011; 2: e176. 10.1038/cddis.2011.57 21697951 PMC3168999

[2] Hwang JJ, Lee SJ, Kim TY et al. Zinc and 4-hydroxy-2-nonenal mediate lysosomal membrane permeabilization induced by H_2_O_2_ in cultured hippocampal neurons. J Neurosci 2008; 28: 3114–22. 10.1523/JNEUROSCI.0199-08.2008 18354014 PMC6670692

[3] Oku Y, Murakami K, Irie K et al. Synthesized Abeta42 caused intracellular oxidative damage, leading to cell death, via lysosome rupture. Cell Struct Funct 2017; 42: 71–9. 10.1247/csf.17006 28413178

[4] Tian Y, Li M, Liu Y. Detection sensitivity enhancement of naphthalimide PET fluorescent probes by 4-methoxy-substitution. Molecules 2020; 25: 4465. 10.3390/molecules25194465 33003286 PMC7582873

[5] Jiang PJ, Guo ZJ. Fluorescent detection of zinc in biological systems: recent development on the design of chemosensors and biosensors. Coord Chem Rev 2004; 248: 205–29. 10.1016/j.cct.2003.10.013

[6] Masanta G, Lim CS, Kim HJ et al. A mitochondrial-targeted two-photon probe for zinc ion. J Am Chem Soc 2011; 133: 5698–700. 10.1021/ja200444t 21449534

[7] Nolan EM, Jaworski J, Okamoto K et al. QZ1 and QZ2: rapid, reversible quinoline-derivatized fluoresceins for sensing biological Zn(II). J Am Chem Soc 2005; 127: 16812–23. 10.1021/ja052184t 16316228 PMC1851667

[8] Qin Y, Dittmer PJ, Park JG et al. Measuring steady-state and dynamic endoplasmic reticulum and Golgi Zn^2+^ with genetically encoded sensors. Proc Natl Acad Sci USA 2011; 108: 7351–6. 10.1073/pnas.1015686108 21502528 PMC3088641

[9] Que EL, Domaille DW, Chang CJ. Metals in neurobiology: probing their chemistry and biology with molecular imaging. Chem Rev 2008; 108: 1517–49. 10.1021/cr078203u 18426241

[10] Tomat E, Nolan EM, Jaworski J et al. Organelle-specific zinc detection using zinpyr-labeled fusion proteins in live cells. J Am Chem Soc 2008; 130: 15776–7. 10.1021/ja806634e 18973293 PMC2645946

[11] Vinkenborg JL, Nicolson TJ, Bellomo EA et al. Genetically encoded FRET sensors to monitor intracellular Zn^2+^ homeostasis. Nat Methods 2009; 6: 737–40. 10.1038/nmeth.1368 19718032 PMC6101214

[12] Xu ZC, Yoon J, Spring DR. Fluorescent chemosensors for Zn^2+^. Chem Soc Rev 2010; 39: 1996–2006. 10.1039/b916287a 20428518

[13] Resch-Genger U, Grabolle M, Cavaliere-Jaricot S et al. Quantum dots versus organic dyes as fluorescent labels. Nat Meth 2008; 5: 763–75. 10.1038/nmeth.1248 18756197

[14] Chen GY, Qju HL, Prasad PN et al. Upconversion nanoparticles: design, nanochemistry, and applications in theranostics. Chem Rev 2014; 114: 5161–214. 10.1021/cr400425h 24605868 PMC4039352

[15] Gao XH, Cui YY, Levenson RM et al. *In vivo* cancer targeting and imaging with semiconductor quantum dots. Nat Biotechnol 2004; 22: 969–76. 10.1038/nbt994 15258594

[16] Hong GS, Antaris AL, Dai HJ. Near-infrared fluorophores for biomedical imaging. Nat Biomed Eng 2017; 1: 0010. 10.1038/s41551-016-0010

[17] Ni DL, Bu WB, Ehlerding EB et al. Engineering of inorganic nanoparticles as magnetic resonance imaging contrast agents. Chem Soc Rev 2017; 46: 7438–68. 10.1039/C7CS00316A 29071327 PMC5705441

[18] Li Y, Hu X, Zhang X et al. Unconventional application of gold nanoclusters/Zn-MOF composite for fluorescence turn-on sensitive detection of zinc ion. Anal Chim Acta 2018; 1024: 145–52. 10.1016/j.aca.2018.04.016 29776540

[19] Ramachandiran D, Rajesh KJMRB. Highly detection of Zn (II) ion sensing and photocatalytic activities of biosynthesized AgNPs using NilgirianthusCiliatus leaf extract and its properties. Mater Res Bull 2022; 149: 111715. 10.1016/j.materresbull.2021.111715

[20] Hu P, Wang R, Zhou L et al. Near-infrared-activated upconversion nanoprobes for sensitive endogenous Zn^2+^ detection and selective on-demand photodynamic therapy. Anal Chem 2017; 89: 3492–500. 10.1021/acs.analchem.6b04548 28220697

[21] Bozym RA, Thompson RB, Stoddard AK et al. Measuring picomolar intracellular exchangeable zinc in PC-12 cells using a ratiometric fluorescence biosensor. ACS Chem Biol 2006; 1: 103–11. 10.1021/cb500043a 17163650

[22] Xue L, Li G, Zhu D et al. Rational design of a ratiometric and targetable fluorescent probe for imaging lysosomal zinc ions. Inorg Chem 2012; 51: 10842–9. 10.1021/ic301307v 23016704

[23] Ripoll C, Martin M, Roldan M et al. Intracellular Zn^2+^ detection with quantum dot-based FLIM nanosensors. Chem Commun 2015; 51: 16964–7. 10.1039/C5CC06676J 26443308

[24] Dey S, Fan C, Gothelf KV et al. DNA origami. Nat Rev Methods Primers 2021; 1: 13. 10.1038/s43586-020-00009-8

[25] Huang Q, Chen B, Shen J et al. Encoding fluorescence anisotropic barcodes with DNA frameworks. J Am Chem Soc 2021; 143: 10735–42. 10.1021/jacs.1c04942 34242004

[26] Yajima S, Koto A, Koda M et al. Photo-cross-linked probe-modified magnetic particles for the selective and reliable recovery of nucleic acids. ACS Omega 2022; 7: 12701–6. 10.1021/acsomega.1c07012 35474845 PMC9026142

[27] Zhou W, Yang F, Li S et al. A stimulus-responsive hexahedron DNA framework facilitates targeted and direct delivery of native anticancer proteins into cancer cells. Chem Sci 2022; 13: 11132–9. 10.1039/D2SC02858A 36320481 PMC9516948

[28] Thekkan S, Jani MS, Cui C et al. A DNA-based fluorescent reporter maps HOCl production in the maturing phagosome. Nat Chem Biol 2018; 15: 1165–72. 10.1038/s41589-018-0176-3 30531966 PMC7034416

[29] Saminathan A, Devany J, Veetil AT et al. A DNA-based voltmeter for organelles. Nat Nanotechnol 2021; 16: 96–103. 10.1038/s41565-020-00784-1 33139937 PMC8513801

[30] Narayanaswamy N, Chakraborty K, Saminathan A et al. A pH-correctable, DNA-based fluorescent reporter for organellar calcium. Nat Meth 2019; 16: 95–102. 10.1038/s41592-018-0232-7 PMC7107693 30532082

[31] Leung K, Chakraborty K, Saminathan A et al. A DNA nanomachine chemically resolves lysosomes in live cells. Nat Nanotechnol 2018; 14: 176–83. 10.1038/s41565-018-0318-5 30510277 PMC6859053

[32] Saha S, Prakash V, Halder S et al. A pH-independent DNA nanodevice for quantifying chloride transport in organelles of living cells. Nat Nanotechnol 2015; 10: 645–51. 10.1038/nnano.2015.130 26098226

[33] Suresh B, Saminathan A, Chakraborty K et al. Tubular lysosomes harbor active ion gradients and poise macrophages for phagocytosis. Proc Natl Acad Sci USA 2021; 118: e2113174118. 10.1073/pnas.2113174118 34607961 PMC8522270

[34] Fang H, Geng S, Hao M et al. Simultaneous Zn^2+^ tracking in multiple organelles using super-resolution morphology-correlated organelle identification in living cells. Nat Commun 2021; 12: 109. 10.1038/s41467-020-20309-7 33397937 PMC7782730

[35] Edwardson TGW, Carneiro KMM, McLaughlin CK et al. Site-specific positioning of dendritic alkyl chains on DNA cages enables their geometry-dependent self-assembly. Nat Chem 2013; 5: 868–75. 10.1038/nchem.1745 24056344

[36] Ge Z, Gu H, Li Q et al. Concept and development of framework nucleic acids. J Am Chem Soc 2018; 140: 17808–19. 10.1021/jacs.8b10529 30516961

[37] Wiraja C, Zhu Y, Lio DCS et al. Framework nucleic acids as programmable carrier for transdermal drug delivery. Nat Commun 2019; 10: 1147. 10.1038/s41467-019-09029-9 30850596 PMC6408537

[38] Manders EMM, Verbeek FJ, Aten JA. Measurement of co-localization of objects in dual-colour confocal images. J Microsc 1993; 169: 375–82. 10.1111/j.1365-2818.1993.tb03313.x 33930978

[39] Li J, Pei H, Zhu B et al. Self-assembled multivalent DNA nanostructures for noninvasive intracellular delivery of immunostimulatory CpG oligonucleotides. ACS Nano 2011; 5: 8783–9. 10.1021/nn202774x 21988181

[40] Mei Q, Wei X, Su F et al. Stability of DNA origami nanoarrays in cell lysate. Nano Lett 2011; 11: 1477–82. 10.1021/nl1040836 21366226 PMC3319871

[41] Liang L, Li J, Li Q et al. Single-particle tracking and modulation of cell entry pathways of a tetrahedral DNA nanostructure in live cells. Angew Chem Int Ed 2014; 53: 7745–50. 10.1002/anie.201403236 24827912

[42] Verwilst P, Kim HR, Seo J et al. Rational design of *in vivo* tau tangle-selective near-infrared fluorophores: expanding the BODIPY universe. J Am Chem Soc 2017; 139: 13393–403. 10.1021/jacs.7b05878 28857559

[43] Koehler D, Shah ZA, Hensley K et al. Lanthionine ketimine-5-ethyl ester provides neuroprotection in a zebrafish model of okadaic acid-induced Alzheimer's disease. Neurochem Int 2018; 115: 61–8. 10.1016/j.neuint.2018.02.002 29475037 PMC5865644

[44] Polanco JC, Li CZ, Bodea LG et al. Amyloid-beta and tau complexity—towards improved biomarkers and targeted therapies. Nat Rev Neurol 2018; 14: 22–39. 10.1038/nrneurol.2017.162 29242522

[45] Kumar S, Henning-Knechtel A, Magzoub M et al. Peptidomimetic-based multidomain targeting offers critical evaluation of a beta structure and toxic function. J Am Chem Soc 2018; 140: 6562–74. 10.1021/jacs.7b13401 29648815

[46] Hanger DP, Anderton BH, Noble W. Tau phosphorylation: the therapeutic challenge for neurodegenerative disease. Trends Mol Med 2009; 15: 112–9. 10.1016/j.molmed.2009.01.003 19246243

[47] van der Kant R, Goldstein LSB, Ossenkoppele R. Amyloid-beta-independent regulators of tau pathology in Alzheimer disease. Nat Rev Neurosci 2020; 21: 21–35. 10.1038/s41583-019-0240-3 31780819

[48] Gowrishankar S, Wu Y, Ferguson SM. Impaired JIP3-dependent axonal lysosome transport promotes amyloid plaque pathology. J Cell Biol 2017; 216: 3291–305. 10.1083/jcb.201612148 28784610 PMC5626538

[49] Ahmad W, Ijaz B, Shabbiri K et al. Oxidative toxicity in diabetes and Alzheimer's disease: mechanisms behind ROS/RNS generation. J Biomed Sci 2017; 24: 76. 10.1186/s12929-017-0379-z 28927401 PMC5606025

[50] Bhatt S, Puli L, Patil CR. Role of reactive oxygen species in the progression of Alzheimer's disease. Drug Discov Today 2021; 26: 794–803. 10.1016/j.drudis.2020.12.004 33306995

[51] Nada SE, Williams FE, Shah ZA. Development of a novel and robust pharmacological model of okadaic acid-induced Alzheimer's disease in zebrafish. CNS Neurol Disord Drug Targets 2016; 15: 86–94. 10.2174/1871527314666150821105602 26295819

[52] van Tijn P, Kamphuis W, Marlatt MW et al. Presenilin mouse and zebrafish models for dementia: focus on neurogenesis. Prog Neurobiol 2011; 93: 149–64. 10.1016/j.pneurobio.2010.10.008 21056616

[53] Kizil C, Iltzsche A, Kaslin J et al. Micromanipulation of gene expression in the adult zebrafish brain using cerebroventricular microinjection of morpholino oligonucleotides. Jove-J Vis Exp 2013; 75: e50415. 10.3791/50415 PMC3718288 23728426

[54] Lin CY, Tseng HC, Chu YR et al. Cerebroventricular injection of Pgk1 attenuates MPTP-induced neuronal toxicity in dopaminergic cells in zebrafish brain in a glycolysis-independent manner. Int J Mol Sci 2022; 23: 4150. 10.3390/ijms23084150 35456967 PMC9025024

[55] Borkowska M, Siek M, Kolygina DV et al. Targeted crystallization of mixed-charge nanoparticles in lysosomes induces selective death of cancer cells. Nat Nanotechnol 2020; 15: 331–41. 10.1038/s41565-020-0643-3 32203435

[56] Zhu HY, Li QQ, Liao TP et al. Metabolomic profiling of single enlarged lysosomes. Nat Meth 2021; 18: 788–98. 10.1038/s41592-021-01182-8 34127857

[57] Williams FE, White D, Messer WS. A simple spatial alternation task for assessing memory function in zebrafish. Behav Proc 2002; 58: 125–32. 10.1016/S0376-6357(02)00025-6 12044689

[58] Wu W-C, Chen C-Y, Tian Y et al. Enhancement of aggregation-induced emission in dye-encapsulating polymeric micelles for bioimaging. Adv Funct Mater 2010; 20: 1413–23. 10.1002/adfm.200902043

[59] Chu Y, Song R, Zhang L et al. Water-dispersible, biocompatible and fluorescent poly(ethylene glycol)-grafted cellulose nanocrystals. Int J Biol Macromol 2020; 153: 46–54. 10.1016/j.ijbiomac.2020.02.286 32112832

[60] Zhang C, Lu T, Tao J et al. Co-delivery of paclitaxel and indocyanine green by PEGylated graphene oxide: a potential integrated nanoplatform for tumor theranostics. RSC Adv 2016; 6: 15460–8. 10.1039/C5RA25518J

[61] Asadi F, Azizi SN, Chaichi MJ. Green synthesis of fluorescent PEG-ZnS QDs encapsulated into Co-MOFs as an effective sensor for ultrasensitive detection of copper ions in tap water. Mater Sci Eng C Mater Biol Appl 2019; 105: 110058. 10.1016/j.msec.2019.110058 31546432

[62] Narayanaswamy N, Chakraborty K, Saminathan A et al. A pH-correctable, DNA-based fluorescent reporter for organellar calcium. Nat Methods 2019; 16: 95–102. 10.1038/s41592-018-0232-7 30532082 PMC7107693

