# Supplementary material for: Targeted imaging of lysosomal zinc ions with a tetrahedral DNA framework fluorescent reporter
Source: Natl Sci Rev. 2024 Sep 12;11(11):nwae307. doi: 10.1093/nsr/nwae307 (PMC11493095; doi:10.1093/nsr/nwae307)
Supplement: nwae307_Supplemental_File [file nwae307_supplemental_file.pdf]

Supplementary Information

Targeted imaging of lysosomal zinc ions with a tetrahedral DNA framework fluorescent reporter

Yue Gao[1,2‡], Xia Liu[3‡], Wei Li[1‡], Yuncong Chen[4], Shitai Zhu[1], Qinglong Yan[3], Shanshan Geng[4], Jichao Zhang[5], Yong Guan[6], Qian Li[7], Sisi Jia[8], Lihua Wang[1,2], Jiang Li[1,2], Weijiang He[4], Chunhai Fan[7]*, Zijian Guo[4]*, Ying Zhu[1,2]*

[1]CAS Key Laboratory of Interfacial Physics and Technology, Shanghai Institute of Applied Physics, Chinese Academy of Sciences, Shanghai 201800, China; University of Chinese Academy of Sciences, Beijing 100049, China

[2]Institute of Materiobiology, College of Science, Shanghai University, Shanghai 200444, China

[3]Xiangfu Laboratory, Jiashan 314102, China

[4]State Key Laboratory of Coordination Chemistry, School of Chemistry and Chemical Engineering, Nanjing University, Nanjing 210023, China

[5]Shanghai Synchrotron Radiation Facility, Shanghai Advanced Research Institute, Chinese Academy of Sciences, Shanghai 201210, China

[6]National Synchrotron Radiation Laboratory, University of Science and Technology of China, Hefei 230026, China

[7]School of Chemistry and Chemical Engineering, New Cornerstone Science Laboratory, Frontiers Science Center for Transformative Molecules and National Center for Translational Medicine, Shanghai Jiao Tong University, Shanghai 200240, China

[8]Zhangjiang Laboratory, 100 Haike Rd, Shanghai 201210, China

[‡]These authors contributed equally: Yue Gao, Xia Liu, Wei Li

*Corresponding author. E-mail: fanchunhai@sjtu.edu.cn, zguo@nju.edu.cn, zhuying331@shu.edu.cn

Supplementary methods

Materials

Chemicals and reagents were obtained from Sigma-Aldrich unless stated otherwise. Single strands alkyl-DNAs were synthesized by Jie Li Biology. LysoTracker®Red was obtained from ThermoFisher Scientific. All the antibodies were purchased from Abcam. Zebrafish were obtained from Shanghai Institutes for Biological Sciences.

Preparation of NapBu-BPEA

4-bromo-N-n-butyl-1,8-naphthalimide (1)[1]:

A mixture of 4-bromo-1,8-naphthalic anhydride (0.661 g, 2.3 mmol) and n-butylamine (0.516 g, 7.05 mmol) in ethanol (30 mL) was refluxed and stirred in 50ml three-necked flask under N2 for 4 hours. After cooling to room temperature, the mixture was filtrated and the residue was washed with ethanol (5 mL ×3), and dried as a light yellow power in 63.4% yield（502mg）. ^{1}H NMR (400 MHz， $CDCl_3$) δ 8.66 (dd, J = 7.3, 1.2 Hz, 1H), 8.57 (dd, J = 8.5, 1.1 Hz, 1H), 8.42 (d, J = 7.9 Hz, 1H), 8.04 (d, J = 7.9 Hz, 1H), 7.85 (dd, J = 8.5, 7.3 Hz, 1H), 4.21 – 4.15 (m, 2H), 1.76 – 1.66 (m, 2H), 1.45 (dq, J = 14.8, 7.4 Hz, 2H), 0.98 (t, J = 7.4 Hz, 3H).

N-n-butyl-4-(aminoethylene)amino-1,8-naphthalimide (2)[1]:

1 g (3.00 mmol) of 4-bromo-N-n-butyl-1,8-naphthalimide and 6.0 mL(90 mmol) ethylenediamine were added in a 10 mL three-necked flask. With continuous stirring at 50 ℃ for 5 h, Then, the mixture was cooled and poured into 100 mL of ice water, and

the precipitate was collected by filtration, washed with water and dried to 64% yield (609mg). ^{1}H NMR (400 MHz, $CDCl_3$) δ 8.59 (dd, J = 7.4, 1.1 Hz, 1H), 8.46 (d, J = 8.4 Hz, 1H), 8.17 (dd, J = 8.6, 1.1 Hz, 1H), 7.62 (dd, J = 8.4, 7.3 Hz, 1H), 6.71 (d, J = 8.4 Hz, 1H), 6.15 (s, 1H), 4.22 – 4.12 (m, 2H), 3.42 (q, J = 5.5 Hz, 2H), 3.18 (dd, J = 6.6, 4.9 Hz, 2H), 1.75 – 1.67 (m, 2H), 1.46 – 1.39 (m, 2H), 0.97 (t, J = 7.3 Hz, 3H).

2,4-(bis(pyridin-2-ylmethyl)aminoethyl)amino-N-n-butyl-1,8-naphthalimide (NapBu-BPEA)[2]:

To a solution of 200 mg (0.64 mmol) N-n-butyl-4-(aminoethylene) amino-1,8-naphthalimide (2) in 20 mL dry ethanol was added 330 mg (2.6 mmol) picolyl chloride and 300 mg K2CO3. The mixture was then heated at reflux for 10 hours under nitrogen and monitored by TLC. After the reaction was completed, the solvent was removed under reduced pressure. The crude product was then purified by alumina column chromatography (CH_2Cl_2:MeOH = 100:1). The compound was obtained as a yellow solid in 24.6% yield (70 mg). ^{1}H NMR (400 MHz, $CDCl_3$) δ 8.81 (dd, J = 8.4, 1.2 Hz, 1H), 8.62 (dd, J = 7.3, 1.1 Hz, 1H), 8.57 (ddd, J = 4.9, 1.8, 0.9 Hz, 2H), 8.41 (d, J = 8.4 Hz, 1H), 7.84 (s, 1H), 7.69 (dd, J = 8.4, 7.3 Hz, 1H), 7.56 (td, J = 7.7, 1.8 Hz, 2H), 7.39 (dt, J = 7.9, 1.1 Hz, 2H), 7.15 (ddd, J = 7.5, 4.9, 1.2 Hz, 2H), 6.53 (d, J = 8.5 Hz, 1H), 4.23 – 4.13 (m, 2H), 4.02 (s, 4H), 3.40 (d, J = 5.5 Hz, 2H), 3.08 (t, J = 5.5 Hz, 2H), 1.76 – 1.65 (m, 2H), 1.51 – 1.36 (m, 2H), 0.97 (t, J = 7.4 Hz, 3H). ^{13}C NMR (101 MHz, $CDCl_3$) δ 164.91, 164.26, 158.46, 150.31, 149.21, 136.72, 134.74, 131.00, 130.03, 127.58, 124.32, 123.43, 122.93, 122.43, 120.79, 109.29, 103.90, 59.70,

51.01, 40.88, 39.94, 30.36, 20.47, 13.93. HRMS (positive mode, m/z): Calcd. 494.2551, found: 494.2647 for $[M+H]^+$。

Determination the detection limit of Znluor$_{ly}$ and NapBu-BEPA

The emission intensity of Znluor$_{ly}$ (1.6 μM, equivalent to 5 μM of NapBu-BEPA) and NapBu-BEPA (5 μM) was collected 8 times to determine the background noise σ(Znluor$_{ly}$) and σ(NapBu-BEPA), respectively[3]. Then the solution was treated with various concentrations of $[Zn^{2+}]_{free}$ from 0-5 μM for Znluor$_{ly}$ and NapBu-BEPA, and all fluorescence spectra were collected after thoroughly mixing. A linear regression curve was then fitted according to the emission intensity in the range of 0-5 μM for Znluor$_{ly}$ and NapBu-BEPA. The detection limit was then determined to be 31.9 nM for Znluor$_{ly}$ and 76.5 nM for NapBu-BEPA (Fig. 3h).

The dissociation constant (K_d) of NapBu-BEPA and Znluor$_{ly}$

K_d was determined by the Benesi-Hildebrand equation[4]

$$\frac{F_0}{F_0 - F} = \frac{F_0}{F_0 - F_{complex}} + \frac{F_0}{F_0 - F_{complex}} * K_d * \frac{1}{M}$$

Where F_0 is the initial fluorescence intensity of a free sensor, $F_{complex}$ is the fluorescence intensity of the Zn^{2+} inclusion complex, F the observed fluorescence intensity at its maximum. and [M] is the metal ion concentration. When $F_0/(F_0-F)$ is plotted against 1/[M], the binding constant is given by the intercept/slope ratio. Upon addition of Zn^{2+} (0, 0.01, 0.02, 0.03, 0.04, 0.05, 0.1, 0.2 μM) to the Znluor$_{ly}$'s solution (1.6 μM, pH 5.0), we recorded the fluorescence emission intensity. From curve fitting

of the fluorescence intensity against the reciprocal of the Zn^{2+} concentration ($1/[Zn^{2+}]$), this Benesi-Hildebrand plot yielded a linear fit, from which the K_d value was estimated to be 20.6 nM for $Znluor_{ly}$ and 113.0 nM for NapBu-BEPA (Fig. 3i).

The loading capability of $Znluor_{ly}$

The single-molecule fluorescence technology was applied to investigate the loading capability of $Znluor_{ly}$[5]. The experiments were conducted on a commercial total internal reflection fluorescence microscopy (TIRFM, N-storm, Nikon) with a high numerical aperture oil immersion 100× objective lens (NA 1.49) and electron multiplying charge coupled devices (EMCCD) camera (Andor, iXon 3). $Znluor_{ly}$ was modified with Cy5. $Znluor_{ly}$-Cy5 was placed on the TIRFM for imaging. Before imaging, $Znluor_{ly}$-Cy5 was purified to remove free fluorescent dyes and Cy5. Then a clean borosilicate coverslip was silanized using (3-Aminopropyl) triethoxysilane for immobilizing $Znluor_{ly}$-Cy5. Briefly, 50 μL of 10 pM $Znluor_{ly}$-Cy5 solution was dropped on the silanized slide and incubated for 30 min, and then rinsed with excess water to remove unbound $Znluor_{ly}$-Cy5. We found that the NapBu-BEPA and Cy5 overlay very well, indicating that the NapBu-BEPA could be encapsulated into the hydrophobic core of TDF-20. Next, single-molecule fluorescence quenching snapshots of the $Znluor_{ly}$-Cy5 were conducted. The $Znluor_{ly}$-Cy5 were first excited with a 405-nm solid state laser for 120 s. The second round of excitation was carried out by using a 647-nm laser for 120 s. The fluorescence intensity kinetics were analyzed from the fluorescence spots across the entire movie through NikonN-STORM analysis

software.

The FRET effect with Cy3-Znluor$_{ly}$

The FRET effect within Znluor$_{ly}$ was used to further verify the encapsulation of NapBu-BEPA molecules in the core of Znluor$_{ly}$. FRET effect can take place between NapBu-BEPA (ex/em: 450/550) and Cy 3(ex/em: 550/570), if their distance is less than 10 nm. One vertex of Znluor$_{ly}$ was modified with Cy3. After encapsulating NapBu-BEPA, FRET effect was detected in fluorescence Spectrometer.

Inductively coupled plasma-mass spectrometry (ICP-MS) determination

TDF-20 was incubated with varying concentrations of Zn^{2+}, separated through ultrafiltration, and subsequently appropriately diluted with 2% nitric acid. The concentration of Zn adsorbed on TDF-20 was determined by ICP-MS (NexION300D, PE-PerkinElmer).

Gel electrophoresis

The success of synthesizing alkyl-DNA and different-shaped DNA framework was confirmed by Native polyacrylamide gel electrophoresis (PAGE), which was performed in 1 x TAE buffer (4 mM Tris base, 2 mM acetic acid, 0.2 mM EDTA) at 4 ℃ for about 1~ 1.5 h. Then gel was stained with GelRed for ~15 min for evaluation purposes[6]. PAGE was conducted with 100 mV for 90 min.

Fluorescence measurement

The Zn^{2+} titration experiments were conducted through F900 fluorescence spectrophotometer (Edinburgh Instruments). 0-15 μM Zn^{2+} were added to the $Znluor_{ly}$'s solution (1.6 μM, pH 5.0) and after thoroughly mixing, fluorescence spectra were collected.

Western blotting analysis

Zebrafish brains were homogenized in protein lysate buffer. Debris was removed by centrifugation and supernatant mixed with SDS-loading sample buffer. Protein samples were analyzed by 10% SDS-PAGE and blotted to PVDF membranes. After washing thrice by PBST (PBS containing 0.1% Tween 20) buffer, the blots blocked for 30 min using 6% nonfat milk and then incubated overnight at 4 ℃ with the primary antibodies as required: Anti-beta Amyloid 1-42(1:1000 dilution, Abcam, ab10148), Anti-Tau (phospho T231) (1:1000 dilution, Abcam, ab151559), and glyceraldehyde 3-phosphate dehydrogenase (GAPDH) (1:1000 dilution, Cell Signaling, 2118S). After washing thrice by PBST, the blots were probed with a goat anti-rabbit/mouse horseradish peroxidase-conjugated antibody (1:1000 dilution, CST, 7074S/7076S) for 1 h. The blots were then developed by incubation with chemiluminescence (ECL) plus and exposed to X-ray film. The densities of all bands were quantified with a computer densitometer (AlphaImagerTM 2200 System Alpha Innotech Corporation, San Leandro, GBBOXchemi-XL1.4). The expression of GAPDH was used as the protein loading control.

Immunostaining

The frozen zebrafish brain sections were placed at room temperature for 30 min. Following twice washing with PBS, they were treated with 0.25% Triton X-100 for 15 min and blocked with PBS containing 6% BSA for 45 min at room temperature. Then, brain sections were co-stained with rabbit Anti-beta Amyloid 1-42(1:200 dilution, Abcam, ab10148) and mouse anti-lysosomal-associated membrane protein 1 (LAMP1, dilution 1:200, Abcam, ab208943) overnight at 4℃. After that, washing with PBS thrice and then the brain sections were co-stained with goat anti-rabbit IgG H&L (Alexa Fluor® 594), goat anti-mouse IgG H&L (Alexa Fluor® 647) for 1h at 37 ℃.

Synchrotron-based X-ray microscopy

High-resolution imaging of cell morphology was performed at beamline BL07W of National Synchrotron Radiation Laboratory (NSRL, Hefei, China). Cell samples were positioned on a silicon nitride window. The images were collected at an X-ray energy of 525 eV.

Synchrotron-based X-ray fluorescence (XRF) microscopy was used to investigate the changes of elemental composition in PC12 cells and zebrafish treated with OKA. The experiments were performed at beamline BL15U1 of Shanghai Synchrotron Radiation Facility (SSRF, Shanghai, China). BL15U1 is a multitechnique hard X-ray microfocus beamline. The K-B mirrors were utilized to focus X-ray to 2 μm, and micro-X-ray fluorescence (μ-XRF) microscopy can measure the composition,

content, and distribution (2D) of elements in samples. The sample was positioned at a 45° angle from the incident X-ray beam, and a silicon-drift detector (SDD) was orientated at a 90° angle from the incident beam to collect the X-ray fluorescence. The incident X-ray energy is chosen as 10keV to excite the X-ray fluorescence of K, Ca, Fe, Cu and Zn elements. The 2D distribution maps of the elements were obtained through raster scanning. The scanning step size was 2 μm and each pixel took 2 seconds. After the 2D mapping, a 30 seconds X-ray fluorescence full spectrum was collected to analyze the content of each element in samples.

Learning and memory function in zebrafish

In brief, after exploring OKA for 0, 3, 9 days, zebrafish were put into two-gallon testing tanks which contained a central white divider and a red card attached to one end of the tank (Fig. 4b). Adjusting the bottom of the divider to allow the zebrafish to swim from one side of the tank to the other. Before the behavioral study, zebrafish had their diet restricted for at least 24 hours and were introduced to the testing tank 48 h prior to any observations. Trials were initiated with a light in the central divider for 3 s. After removing the light for 5 s, presenting food originally at the side of the tank with the red card cue, then at the side opposite the card for a period of 28 trials. To avoid zebrafish satiation and complacency, only a small amount of food was offered each time. A correct response was defined as the physical presence of the animal on the side of the tank used for food presentation during that trial. Observations at all three time points (at the cue, 5 s after the cue during food presentation (choice stage),

and 5 s after the food presentation) were recorded and correct responses were compiled for averages and statistical purposes.

Analysis of Zebrafish learning and memory

Zebrafish learning and memory was analyzed according to Smith's method[7]. The equation of the probability, P, of a correct response is:

$$\mathrm{p} = 0.5 + \frac{b\left({}^{t}/_{c}\right)^{5}}{1 + \left({}^{t}/_{c}\right)^{5}}$$

where "b" represents the amount of learning, "c" represents the number of trials it takes to reach half-maximum learning, and "t" is the trial number. The SAS nonlinear modeling procedure NLIN was used to measure the parameters "b" and "c". The parameter "b" will be referenced in the paper as "maximum learning". Plots of the final prediction for P and the success frequency as functions of trial for each treated group were overlaid and used to display the fit of the estimated model.

Supplementary figures and tables.

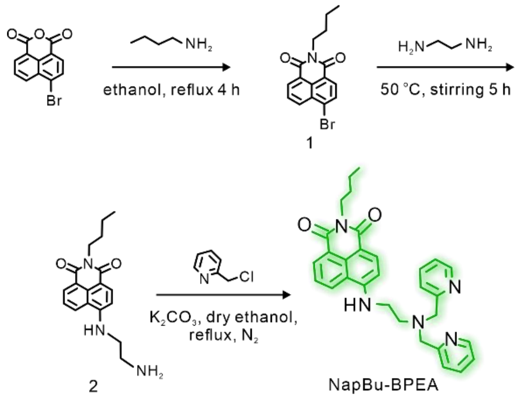

Supplementary Figure 1. Synthetic routes of NapBu-BPEA.

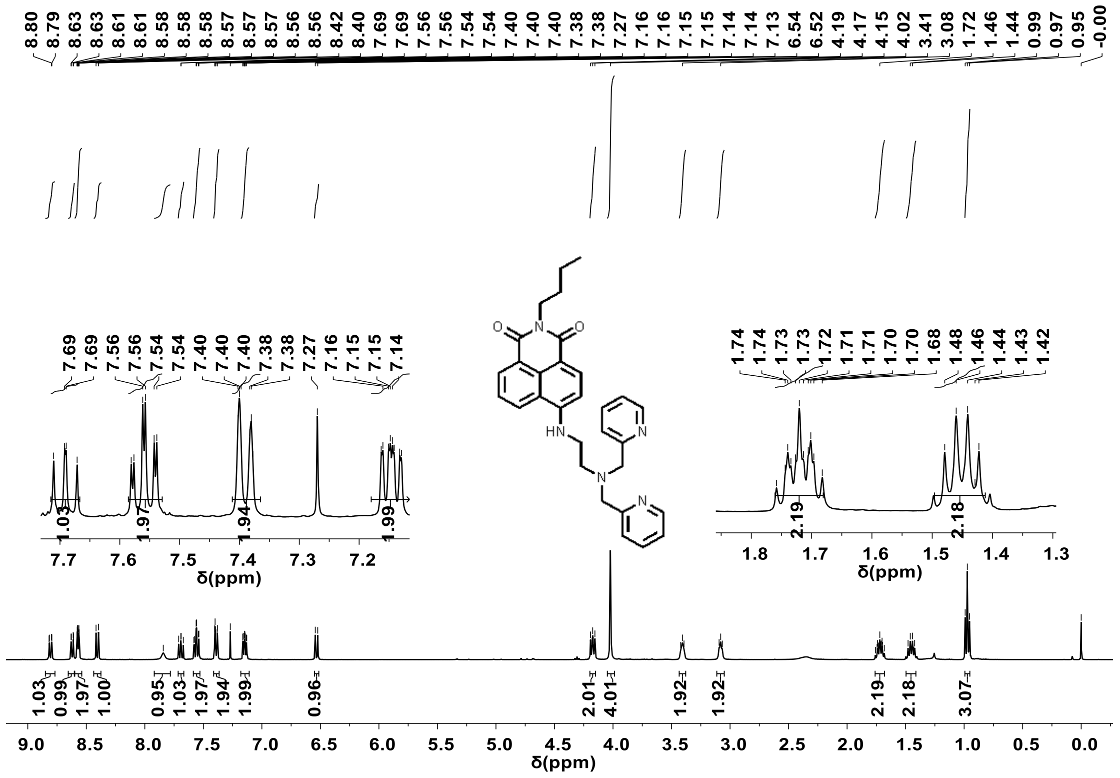

Supplementary Figure 2. ^{1}H NMR spectra of NapBu-BPEA in $CDCl_3$.

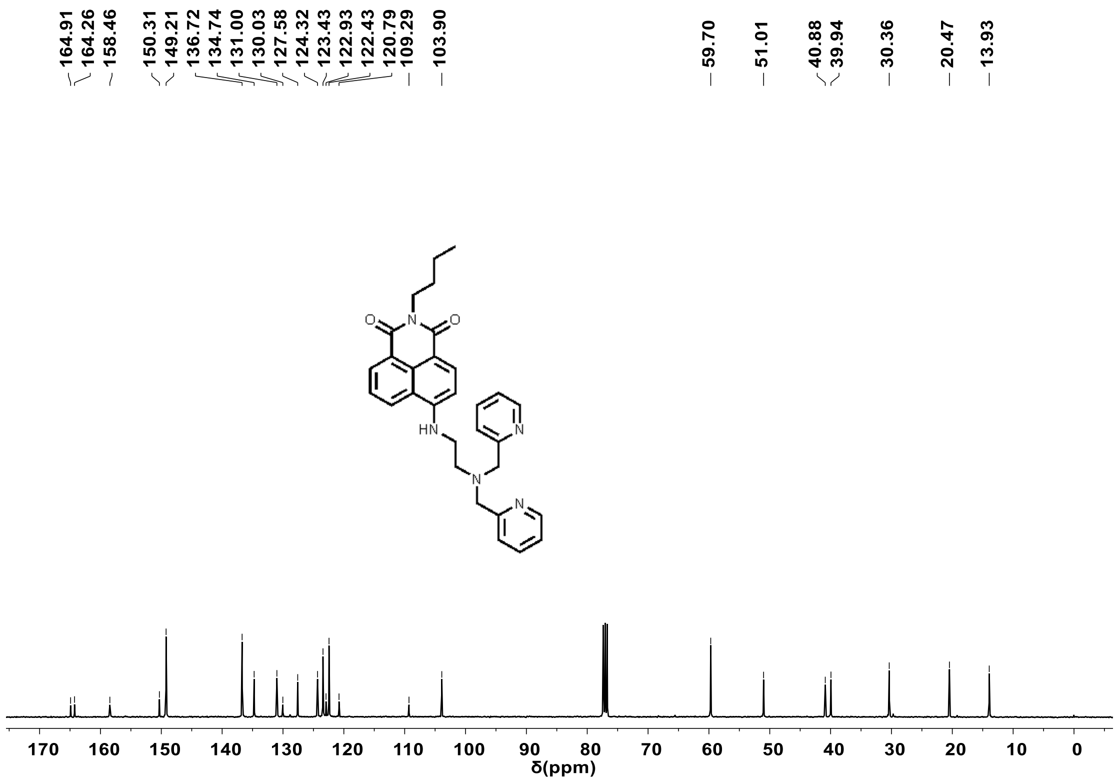

Supplementary Figure 3. ^{13}**C NMR spectrum of NapBu-BPEA in** $CDCl_3$**.**

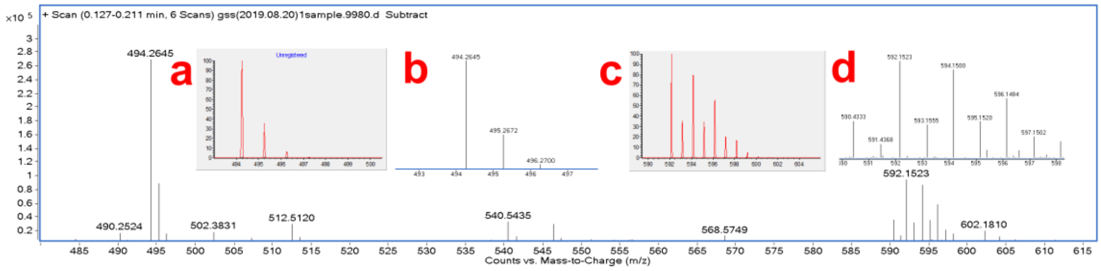

Supplementary Figure 4. HRMS of NapBu-BPEA/Zn^{2+} complex (positive mode). The determined isotopic distribution patterns (IDPs) of the peaks with m/z of 494.2645 and 592.1523 were shown as insets **a** and **c**. The related simulated IDPs for $[M+H]^+$ and $[M+Zn+Cl]^+$ were shown as insets **b** and **d**.

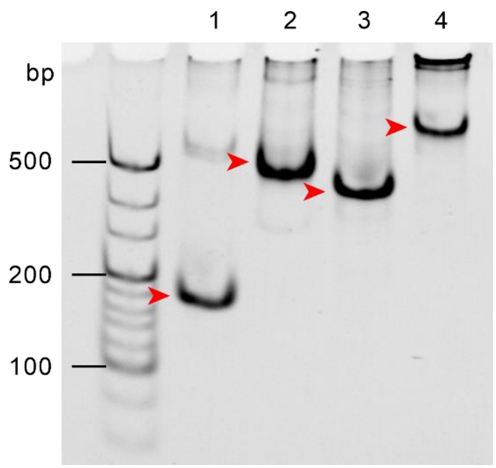

Supplementary Figure 5. Native polyacrylamide gel electrophoresis (PAGE) of different DNA frameworks. Ladder: DNA marker (20 bp). Lane 1: TDF-20. Lane 2: Cube-20. Lane 3: TP-20. Lane 4: TDF-37.

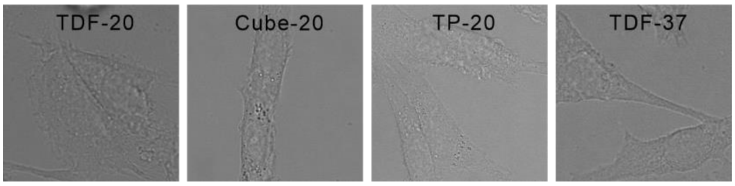

Supplementary Figure 6. Brightfield imaging of Figure 2c.

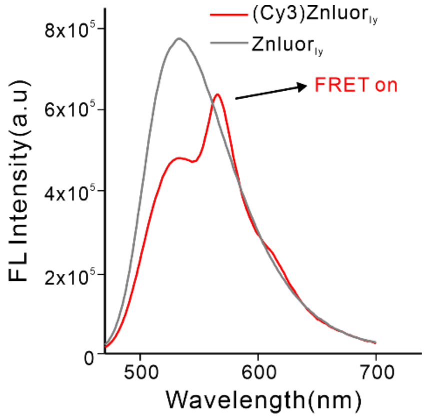

Supplementary Figure 7. The FRET effect took place within Znluor$_{ly}$, indicating the success of encapsulation of NapBu-BEPA into TDF-20. Cy3 (λ_{ex}=561) was chosen to modify Znluor$_{ly}$.

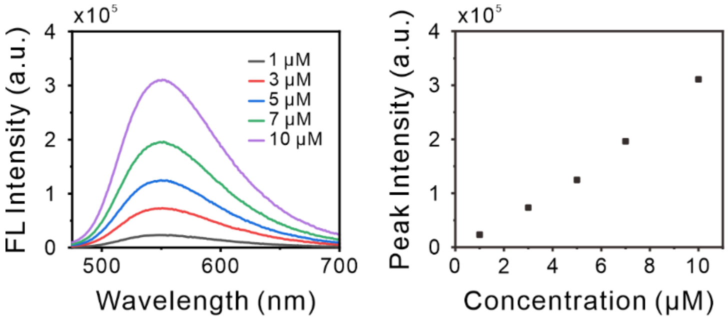

Supplementary Figure 8. Fluorescence spectra of NapBu-BPEA in TM buffer. NapBu-BPEA have no quenching point within the tested concentration range.

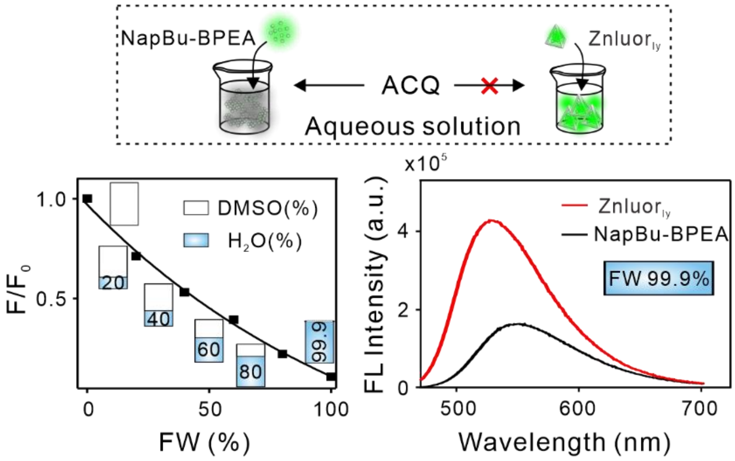

Supplementary Figure 9. ACQ effect of NapBu-BPEA (5 μM) in aqueous solution (left): the fluorescence intensity decreased with increasing fraction of water. The comparison of the fluorescence intensity of Znluor$_{ly}$ (1.6 μM) and NapBu-BPEA (5 μM) in 99.9% fraction of water (FW) (right).

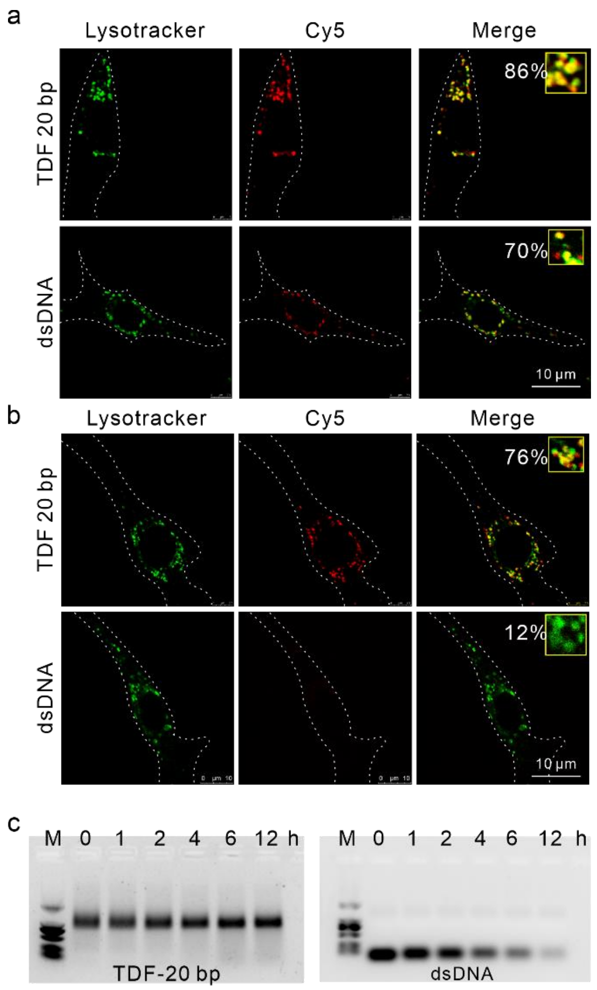

Supplementary Figure 10. Comparison of stability between TDF-20 and dsDNA. (a, b) Lysosomal co-localization at different times (a) 2 h and (b) 6 h. (c) TDF-20 and dsDNA were incubated in a medium with 10% FBS for 0 - 12 h and then analyzed with 1% agarose gel electrophoresis (AGE).

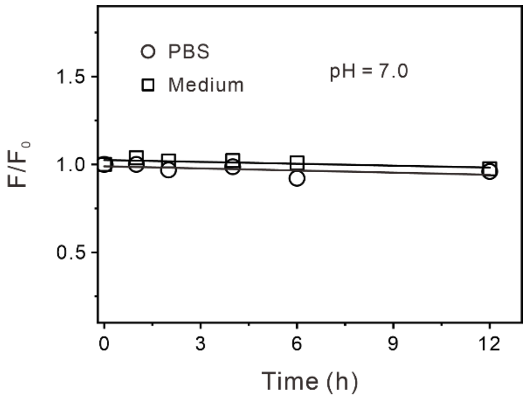

Supplementary Figure 11. The stability of Znluor$_{ly}$ in PBS and Medium at pH = 7.0. The fluorescence Znluor$_{ly}$ keeps stale in both PBS and Medium.

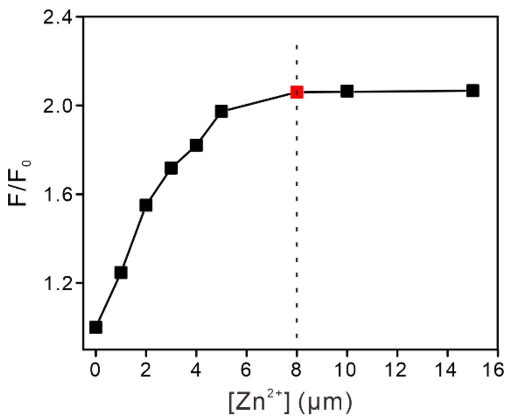

Supplementary Figure 12. Changes of intensity plots of Znluor$_{ly}$ under different concentrations of Zn^{2+}.

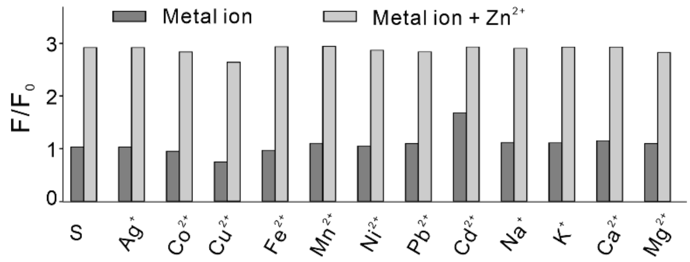

Supplementary Figure 13. Fluorescence intensity of Znluor$_{ly}$ (1.6 μM) in TM buffer in the presence of 1 equiv. Znluor$_{ly}$ sensor (s) or Ag^+, Co^{2+}, Cu^{2+}, Fe^{2+}, Mn^{2+}, Ni^{2+}, Pb^{2+}, Cd^{2+}, or 1000 equiv. Na^+, K^+, Ca^{2+}, Mg^{2+}. Gray bars, the ratio in the presence of Zn^{2+} (1 equiv.), or the indicated metal ions (1 equiv.) followed by adding 1 equiv. Zn^{2+}.

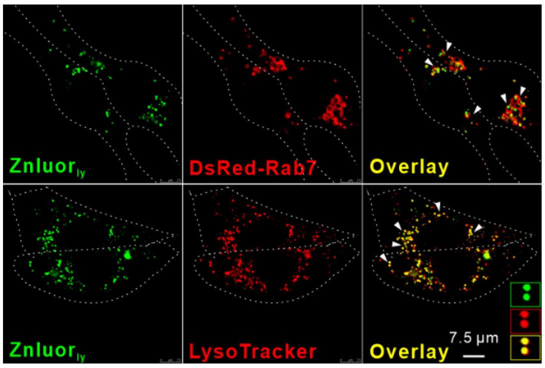

Supplementary Figure 14. Trafficking of Znluor$_{ly}$ in PC12 expressing DsRed-Rab7 (upper), or Lysotracker Red (lower) at the indicated times. Znluor$_{ly}$-positive vesicles are shown in green and DsRed-Rab7/lysotracker vesicles are shown in red. Scale bars, 7.5 μm.

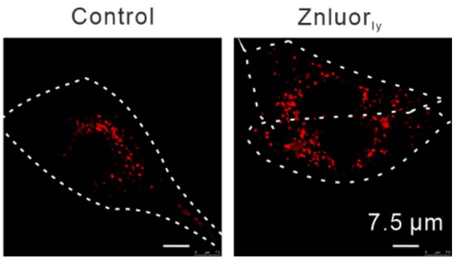

Supplementary Figure 15. Fluorescence imaging of lysosomes before and after Znluor$_{ly}$ treatment. Pseudocolored representative images were generated using Image J. Scale bar: 7.5 μm.

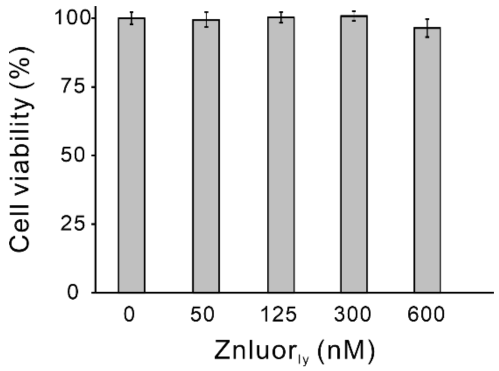

Supplementary Figure 16. Effect of different concentrations of Znluor$_{ly}$ on cell viability. Cell viability was assessed by MTT assay.

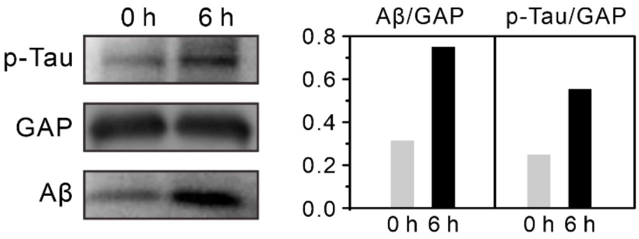

Supplementary Figure 17. Western blot analysis of Aβ and p-Tau. PC12 cells were pre-treated with 100 nM OKA for 0 h or 6 h.

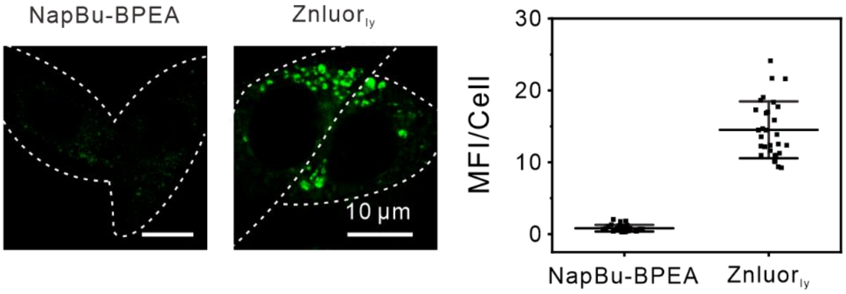

Supplementary Figure 18. Lysosomal endogenous Zn^{2+} detection in live AD cells. Right: Confocal fluorescence imaging of PC12 cells stained with NapBu-BPEA (300 nM, 2 h) and $Znluor_{ly}$ (300 nM, 2 h) at 37 °C, after the cells were treated with OKA for 4 h. Left: The fluorescence quantification and statistics were carried out using Image J. Data are shown individually and as the mean ± S.D. (centre line with error bar). Scale bar: 10 μm.

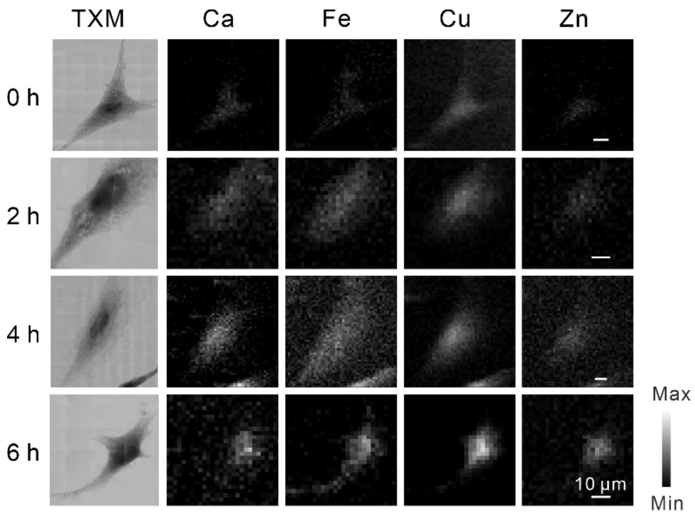

Supplementary Figure 19. The content of other ions in AD cells was measured by TXM and μXRF.

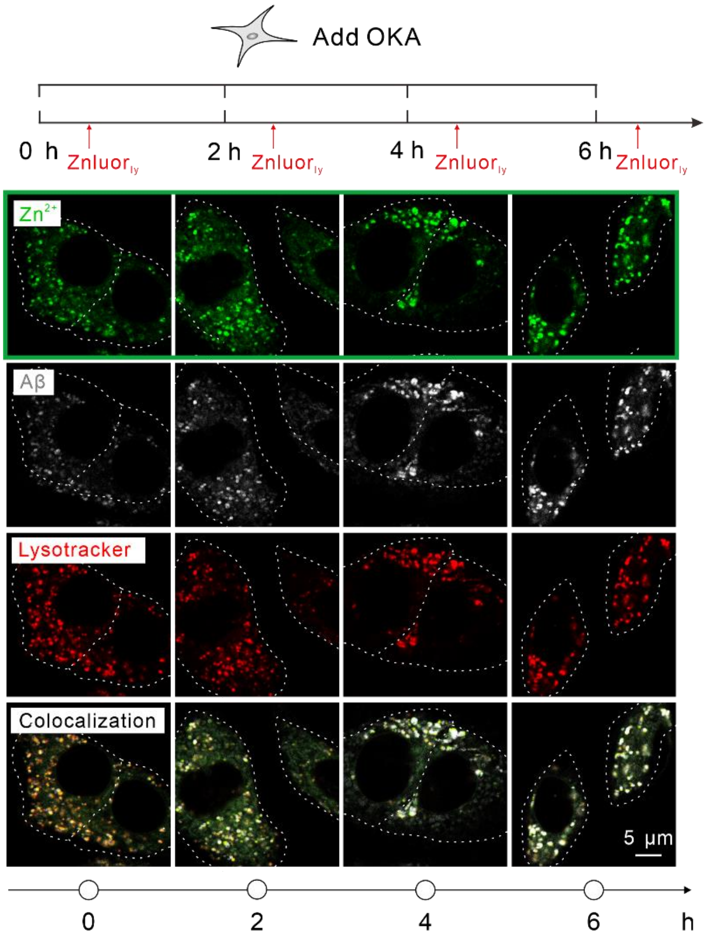

Supplementary Figure 20. **PC12 cells were pre-treated with 100 nM okadaic acid (OKA) at various times, and then stained with Znluor$_{ly}$ (300 nM), thioflavin T (Th T, 300 nM), and LysoTracker Red (a commercially available marker for lysosomes; 1:20000) for 2 h.** Pseudocolored representative images were generated using Image J. Scale bar: 5 μm.

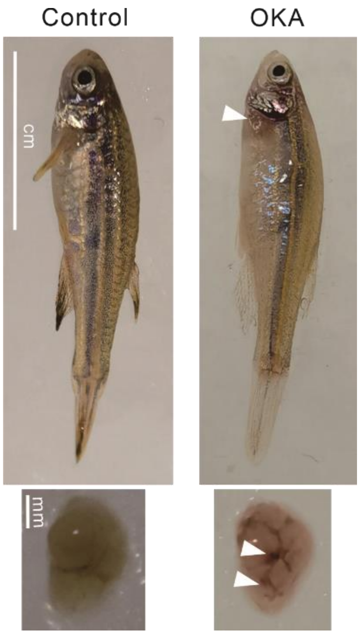

Supplementary Figure 21. Morphological changes induced by OKA. There are microbleeds in the periphery (scale bar=1 cm) and brain (scale bar=1 mm) of zebrafish.

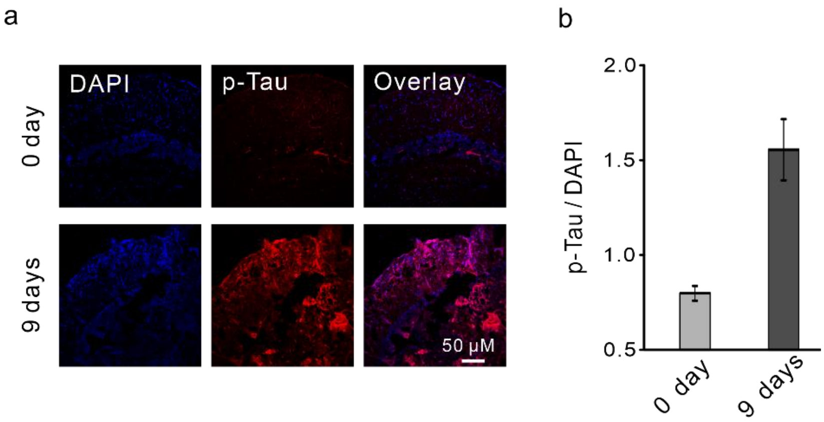

Supplementary Figure 22. Confocal imaging of brain of an OKA treated Zebrafish. The brain slice was incubated with p-Tau primer antibody. After washing with PBS, the brain slice was stained with goat anti-rabbit IgG H&L (Alexa Fluor® 594).

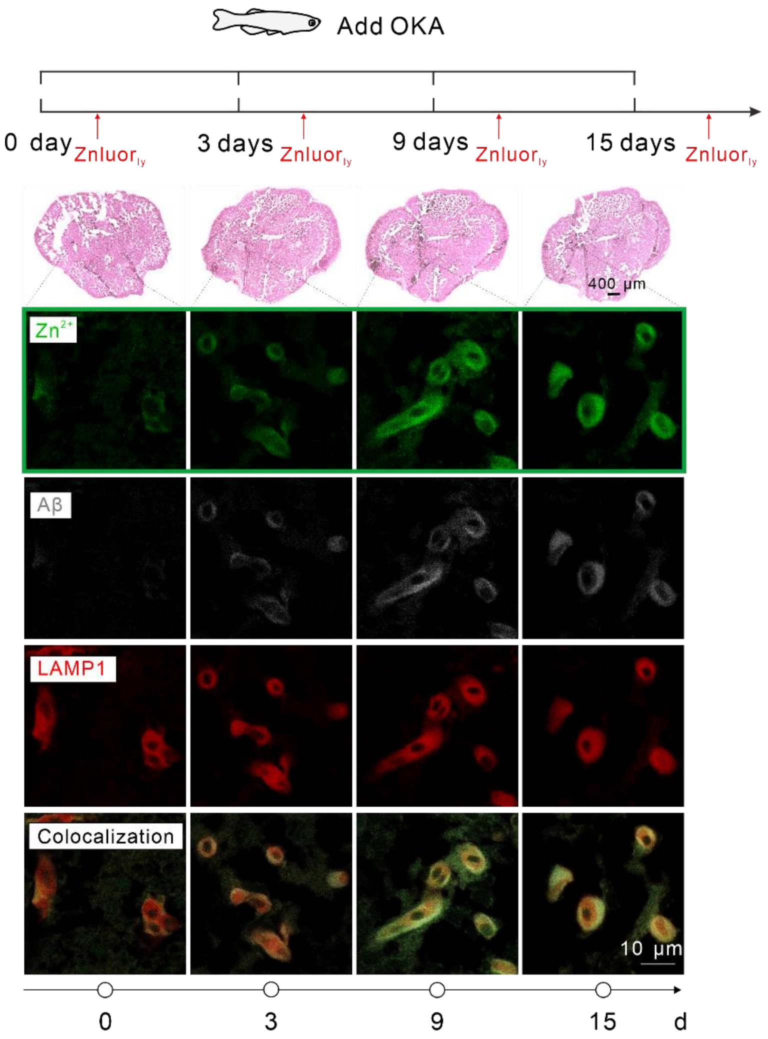

Supplementary Figure 23. Zebrafish were injected with Znluor$_{ly}$ for 2 h (300 nM) and then the brain slice sections were obtained and stained with Aβ antibody (1:1000) and lysosomal associated membrane protein 1 (LAMP1) for 1 h. Pseudocolored representative images were generated using Image J. Scale bar: 10 μm.

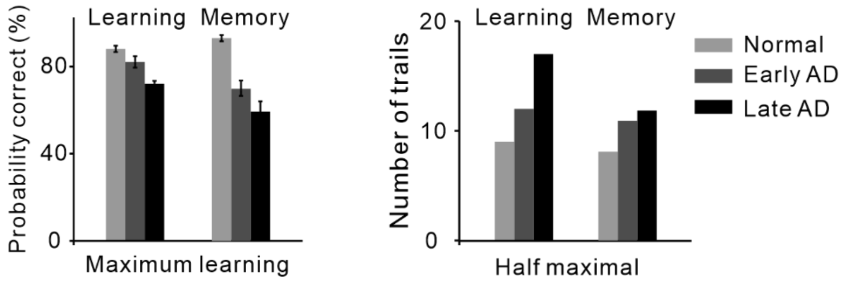

Supplementary Figure 24. Learning and memory data of AD zebrafish.

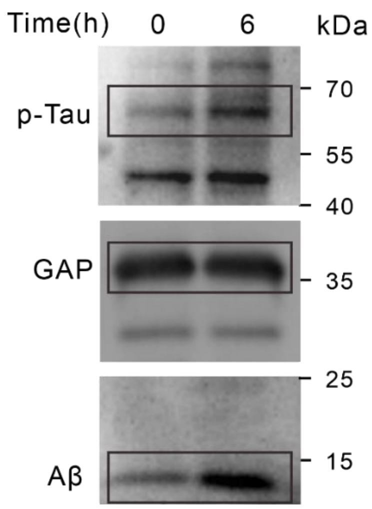

Supplementary figure 25. Uncropped scans of western blots included in Supplementary figure 17.

Table. 1. DNA sequences and chemical structure of dendritic alkyl chains used in this work.

Tetrahedron (20 bp)

Strands	Sequence (5'-xx-3')	Length (bps)
S1	D-TTTCAGGCAGTTGAGACGAACATTCCTAAGTCTGAAATTTATCACCCGCCATAGTAGACGTATCAC	66
S2	D-TTTAGCTTGCTACACGATTCAGACTTAGGAATGTTCGACATGCGAGGGTCCAATACCGACGATTAC	66<br>66
S3	D-TTTGCGGGTGATAAAACGTGTAGCAAGCTGTAATCGACGGGAAGAGCATGCCCATCCACTACTATG	66
S4	D-TTTGGACCCTCGCATGACTCAACTGCCTGGTGATACGAGGATGGGCATGCTCTTCCCGACGGTATT	66

Tetrahedron (37 bp)

Strands	Sequence (5'-xx-3')	Length (bps)
A-5	D-CCCTGTACTGGCTAGGAATTCACGTTTTAATCTGGGCTTTGGGTTAAGAAACTCCCCG	58
A-3	CGCTGGAGGCGCATCACCGTTTGCGTATGTGTTCTGTGCGGCCTGCCGTCCCGTGTGGG	59
B-5	D-CGGTGATGCGCCTCCAGCGCGGGGAGTTTCTTAACCCTTTCCGACTTACAAGAGCCGG	58
B-3	GCGAGACTCAGGTGGTGCCTTTGGCATTCGACCAGGAGATATCGCGTTCAGCTATGCCC	59
C-5	D-CCCATGAGAATAATACCGCCGATTTACGTCAGTCCGGTTTCCCACACGGGACGGCAGGC	59
C-3	CGCACAGAACACATACGCTTTGGGCATAGCTGAACGCGATATCTCCTGGTCGAATGCC	58
D-5	D-GCCCAGATTAAAACGTGAATTCCTAGCCAGTACAGGGTTTCCGGACTGACGTAAATCGG	59
D-3	CGGTATTATTCTCATGGGTTTGGCACCACCTGAGTCTCGCCCGGCTCTTGTAAGTCGG	58

Cube (20 bp)

Strands	Sequence (5'-xx-3')	Length (bps)
1AA	TCGCTGAGTATTTTTCCTATATGGTCAACTGCTCTTTTGCAAGTGTGGGCACGCACACTTTTT<br>CCTATATGGTCAACTGCTCTTTTCACAAATCTG	96
2AA	CTATCGGTAGTTTTTCCTATATGGTCAACTGCTCTTTTTACTCAGCGACAGATTTGTGTTTTT<br>CCTATATGGTCAACTGCTCTTTTCAACTAGCGG	96
3AA	CACTGGTCAGTTTTTCCTATATGGTCAACTGCTCTTTTCTACCGATAGCCGCTAGTTGTTTTT<br>CCTATATGGTCAACTGCTCTTTTGGTTTGCTGA	96
4AA	CCACACTTGCTTTTTCCTATATGGTCAACTGCTCTTTTCTGACCAGTGTCAGCAAACCTTTTT<br>CCTATATGGTCAACTGCTCTTTTGTGTGCGTGC	96
A'	D-TTTTTCAGTTGACCATATA	19

Triangular Prism (20 bp)

Strands	Sequence (5'-xx-3')	Length (bps)
1aa	TCGCTGAGTATTTTGCCTGGCCTTGGTCCATTTGTTTTGCAAGTGTGGGCACGCACACTTTT<br>GCCTGGCCTTGGTCCATTTGTTTTCACAAATCTG	96
2aa	CACTGGTCAGTTTTGCCTGGCCTTGGTCCATTTGTTTTTACTCAGCGACAGATTTGTGTTTT<br>GCCTGGCCTTGGTCCATTTGTTTTGGTTTGCTGA	96
3aa	CCACACTTGCTTTTGCCTGGCCTTGGTCCATTTGTTTTCTGACCAGTGTCAGCAAACCTTTT<br>GCCTGGCCTTGGTCCATTTGTTTTGTGTGCGTGC	96
a'	D-CAAATGGACCAAGGCCAGGC	20

Dendritic alkyl chain (D)=

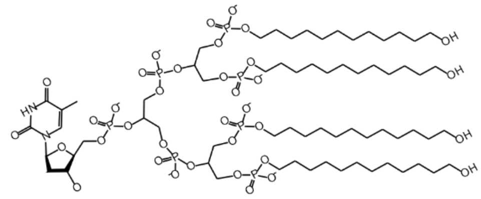

Reference:

1. Qin J-C, Yan J, Wang B-d *et al*. Rhodamine–naphthalene conjugate as a novel ratiometric fluorescent probe for recognition of Al3+. *Tetrahedron Lett*. 2016; **57**(17): 1935-1939.
2. Kawabata E, Kikuchi K, Urano Y *et al*. Design and synthesis of zinc-selective chelators for extracellular applications. *J Am Chem Soc*. 2005; **127**(3): 818-819.
3. Liu Z, Zhang C, Chen Y *et al*. In vivo ratiometric Zn2+ imaging in zebrafish larvae using a new visible light excitable fluorescent sensor. *Chem Commun (Camb)*. 2014; **50**(10): 1253-1255.
4. Indirapriyadharshini VK, Karunanithi P, Ramamurthy P. Inclusion of Resorcinol-Based Acridinedione Dyes in Cyclodextrins: Fluorescence Enhancement. *Langmuir*. 2001; **17**(13): 4056-4060.
5. Li J, Dai JB, Jiang SX *et al*. Encoding quantized fluorescence states with fractal DNA frameworks. *Nat Commun*. 2020; **11**(1): 10.
6. Wiraja C, Zhu Y, Lio DCS *et al*. Framework nucleic acids as programmable carrier for transdermal drug delivery. *Nat Commun*. 2019; **10**(1): 1147.
7. Smith LE, Iii MJC, Dellinger JA *et al*. Developmental selenomethionine and methylmercury exposures affect zebrafish learning. *Neurotoxicol Teratol*. 2010; **32**(2): 246-255.
